# Advanced prediction and optimization of VCR engine characteristics using RSM with DFA for sustainable biofuel derived from waste lemon Peel

**DOI:** 10.1038/s41598-025-20103-9

**Published:** 2025-10-06

**Authors:** Thota S S Bhaskara Rao, Rajayokkiam Manimaran, C. Manivannan, Vallapureddy Siva Nagi Reddy, Kulmani Mehar

**Affiliations:** 1 Department of Mechanical Engineering, Madanapalle Institute of Technology & Science, Madanapalle, Andhra Pradesh India; 2 School of Mechanical Engineering, SRM Institute of Science and Technology, Tiruchirappalli campus, Tiruchirappalli, Tamil Nadu India; 3 Department of Chemistry, SRM Institute of Science and Technology, Tiruchirappalli campus, Tiruchirappalli, Tamil Nadu India; 4 Department of Mechanical Engineering, Aditya University, Surampalem, Andhra Pradesh India; 5 Department of Mechanical and Industrial Engineering, Manipal Institute of Technology, Manipal Academy of Higher Education, Manipal, India

**Keywords:** Lemon peel waste oil, LPWO blends, RSM-CCD prediction, DFA optimization, VCR engine characteristics, Energy science and technology, Engineering, Environmental sciences

## Abstract

The rising demand for alternative fuels stems from fossil fuel depletion, rising crude oil prices, and environmental concerns. Diesel engines, valued for efficiency and durability, contribute to resource depletion and pollution. Biofuels offer a sustainable alternative, with waste lemon peels presenting a viable feedstock for biofuel production. Using a steam distillation process, lemon peel waste oil (LPWO) is extracted from waste lemon peels and test fuel blends of LPWO and conventional diesel have been created in ratios of 5%, 10%, 15%, and 20%. According to the ASTM standards, the properties of LPWO and its blends, along with diesel, have been assessed. The characteristics of LPWO were determined by FTIR, GC-MS, and TG/dTG analysis. The performance, combustion, and emission parameters have been evaluated for neat LPWO and LPWO blends in a variable compression ratio (VCR) engine by varying BP between 0 kW and 5.2 kW and compression ratio from 16:1 to 18:1. From experimental analysis, optimum results are observed while using the blend 5% LPWO, BP 5.2 kW and CR 18:1. LPWO5 showed an increase in BTE and EGT by 2.168% and 3.09% while minimizing BSFC by 6.54%, also improved HRR and in-cylinder pressure; a decrease of CO, NO_x_, and smoke emissions by 59.42%, 30.99%, and 7.89% whereas 9.14% and 0.201% increase in HC and CO_2_ when compared to diesel fuel. To model and optimize the engine responses, a multiple regression model was developed using response surface methodology (RSM) with a desirability function approach (DFA). The optimal operating conditions predicted were 6.51% LPWO blend, 1.42 kW load, and CR 18:1, which closely aligned with experimental findings. The RSM-CCD design coupled with the DFA model yielded a combined desirability value of 0.8997. The VCR engine results were validated with the RSM predictions and DFA optimization, showing an error margin of less than 5%. These outcomes indicate that the LPWO5 blend holds strong potential as a viable alternative fuel for VCR engine applications.

## Introduction

 Fossil fuels, derived from coal, oil, and natural gas, serve as primary non-renewable energy sources. Diesel fuel, a petroleum-derived hydrocarbon mixture, is obtained through the fractional distillation of crude oil^1^. Due to its higher boiling point, diesel exhibits more excellent thermal stability than gasoline. It is predominantly utilized in compression ignition (CI) engines, compressing air to elevate its temperature, facilitating spontaneous diesel combustion and efficient energy conversion^2^. While diesel engines offer superior fuel and thermal efficiency over spark ignition (SI) engines, their increasing demand has escalated petroleum prices. Additionally, diesel combustion releases nitrogen oxides (NOₓ) and particulate matter (PM), contributing to environmental pollution and adverse health effects^3^. Researchers are actively exploring alternative fuels to reduce exhaust emissions and decrease reliance on conventional energy sources, addressing environmental challenges caused by fossil fuel consumption^4^. Viable alternatives such as biogas, ethanol, compressed natural gas (CNG), and various biofuels have been extensively studied. Biofuels, derived from renewable natural sources, are gaining popularity due to their widespread availability, improved efficiency, environmental sustainability, and economic feasibility^5^. These fuels are obtained from diverse sources, including plants, trees, animal fats, and biomass from agricultural, industrial, and domestic waste^6^. In recent years, the production of renewable biofuels from different tree and plant derivatives has increased, with notable properties such as higher net calorific value, lower viscosity, and greater density^7^. Common biofuels include orange oil, sunflower oil, eucalyptus oil, soybean oil, lemon peel oil, and pine oil. Experimental studies have demonstrated the feasibility of biofuels as partial diesel substitutes in CI engines^8^.

LPWO is a promising alternative for diesel engine applications due to its abundant availability, renewable nature, simple extraction process, and low viscosity. Brazil, Mexico, Argentina, and India collectively produce over half of the world’s lemons. Known for its sour taste and nutritional benefits, lemon is among the most widely consumed fruits globally^9^. The large-scale production of lemon juice generates substantial amounts of wet solid waste, with lemon peels constituting the majority. These peels contain numerous oil-bearing glands rich in citrus oil^10^. Instead of being discarded as waste, lemon peels can be utilized for oil extraction. The composition of lemon peel oil varies based on the type of lemons cultivated in different regions. It is typically extracted from waste lemon peels using steam distillation^11^. Dhana Raju et al. ^12^ analyzed the physical and chemical properties of lemon peel oil (LPO) and its diesel blends. The study found that a 20% LPO blend (LPO20) improved performance and reduced emissions. Adding 10% diethyl ether to LPO20 increased brake thermal efficiency (BTE) by 3.7%, while HC, CO, smoke, and NOₓ emissions decreased by 24.4%, 16.9%, 11.8%, and 12.5%, respectively. DIESEL-RK modelling showed slight increases in BTE (2.51%), CP (1.2%), and NOₓ (2.24%) at full load. Overall, the study highlights LPO’s potential to enhance engine performance and lower emissions. Ashok and Thundil Karuppa Raj^13^ found that LPO has a lower viscosity, flash point, and boiling point than diesel. The LPO-diesel blends (20%, 40%, 50%, and 100%) were tested and observed that a diesel engine running on 100% LPO achieved 12% higher BTE and 9% lower brake-specific fuel consumption (BSFC) at peak load compared to diesel. While BSCO, BSHC, and smoke emissions decreased significantly, BSNO_x_ emissions increased by 55%. LPO and its blends caused longer ignition delays, an increase in in-cylinder pressure and heat release. At 200 bar injection pressure and 23° before TDC injection timing, 100% LPO improved engine performance but led to higher vibrations and NOₓ emissions. Udayakumar et al.^14^ analyzed diesel engine performance using B10, B20, and B30 lemon peel biodiesel blends with thermal barrier coatings like yttria-ceria-stabilised zirconia and ceria nanoparticle additives. Results showed improved volumetric efficiency, reduced friction power, and minimal differences in CO and CO₂ emissions between coated and uncoated engines, highlighting the potential of LPO biodiesel with additives in diesel applications.

Ashok et al.^11^ evaluated a 20% LPO blend in diesel under idling conditions. At optimal idle settings, smoke and CO emissions decreased by 42.1% and 16%, while CO₂ increased by 5.25%. Due to its lower heating value, thermal efficiency dropped by 10.18%, and specific fuel consumption rose by 21.24%. The blend exhibited cleaner combustion and improved combustion characteristics. Khan et al.^15^ optimized diesel engine performance using biodiesel, cerium oxide nanoparticles, and hydrogen, analyzed via RSM. The optimal parameters of 28.68% biofuel blend, 87.88% load, 80 ppm NPC, CR 19, and 194.54 bar injection pressure resulted in 33.57% BTE, 0.2550 BSEC, 461.3 ppm NOₓ, 28.08 ppm HC, and a 22.21% vibration reduction. Vellaiyan and Amirthagadeswaran^16^ emulsified lemon peel oil with water and Sorbian mono-laurate using mechanical homogenization for fuel preparation. They tested the fuel in a single-cylinder, four-stroke, air-cooled diesel engine at various brake mean effective pressure settings. The results showed that lemon peel oil enhanced BSFC by 11% and BTE by 16%, while reducing HC, CO, and smoke emissions. Specifically, the LPO10W emulsion reduced HC, CO, and NOx emissions by 18.7%, 33.3%, and 26%, respectively, and improved BSFC and BTE by 9.8% and 11.6%. Ashok et al.^17^ extracted lemon essential oil via steam distillation and compared its properties with diesel. A 20% blend (LEO20) was tested for engine performance, showing reduced smoke, unburned hydrocarbons, and CO emissions, with a slight increase in brake thermal efficiency. However, NOx emissions were higher at rated power. Heat release and cylinder pressure remained comparable to diesel, suggesting LEO20 as a potential partial diesel substitute. Bragadeshwaran et al.^18^ studied lemon peel oil (LPO) emulsions with diesel for biodiesel production, using Span80 and methyl-dihydroxy propyl imidazolium chloride as surfactants. Among the four samples, LPO2 and LPO4 remained stable after seven days. These showed a 5% drop in brake thermal efficiency (BTE) and a 15% reduction in NOx emissions, but over a 50% increase in CO and a significant rise in HO emissions compared to pure LPO. Vellaiyan and Kandasamy^19^ analyzed the impact of cetane improver and water emulsion on diesel engines using lemon peel oil. They evaluated a four-stroke diesel engine’s performance, emissions, and cost-effectiveness. The study compared brake thermal efficiency, brake-specific energy and fuel consumption, and emissions against pure diesel, including a cost-benefit analysis. Bragadeshwaran et al.^20^ studied different lemon peel oil-diesel blends, focusing on efficiency and emissions. Advancing fuel injection to 25° and 27° before TDC improved efficiency, peaking at 6% at 27°. Combined with exhaust gas recirculation, this approach reduced emissions by 45%.

RSM is a statistical tool widely used for predicting and optimizing diesel engine characteristics. By systematically varying input parameters like fuel blend ratio, injection timing, CR, and EGR rate, RSM develops quadratic regression models that capture their effects on outputs, including performance and exhaust emissions^21^. Using CCD or BBD designs with analysis of variance (ANOVA) validation, RSM reduces the number of experiments while ensuring accurate prediction of parameter interactions. 3D surface plots and desirability functions further help identify optimal operating conditions^22^. There are numerous research works on RSM with DFA applications for the prediction and optimization of unmodified diesel engine characteristics with different biodiesel blends, as listed in Table 1.

### Novelty and research objective of the study

Among the various biofuel options for diesel engine applications based on the above discussions, LPWO is considered a promising alternative. Moreover, no research work has yet been reported on the application of RSM combined with DFA for predicting and optimizing VCR engine characteristics using LPWO/diesel blends. Therefore, this study aims to evaluate the viability of utilizing LPWO blends as a substitute for conventional diesel fuel in a VCR engine. The research involves extracting LPWO from wasted lemon peels using the steam distillation process and preparing LPWO5, LPWO10, LPWO15, and LPWO20 blends. The properties of these blends, including density, flash and fire points, and calorific values, will be analyzed in accordance with ASTM standards. Additionally, their chemical characteristics will be assessed using GC-MS, FTIR, and TGA analysis and compared with conventional diesel. The study further investigates the performance, combustion, and emission characteristics of LPWO and its blends under varying loads and compression ratios ranging from 16:1 to 18:1. A multiple regression model will be developed using RSM with DFA to predict and enhance the performance of LPWO blends in a VCR engine. Finally, the optimal LPWO blend will be selected based on experimental and optimization results for potential use as a substitute fuel in VCR engine applications.

**Table 1:** Studies on RSM with DFA applications for prediction and optimization of unmodified diesel engine characteristics with biodiesel blends.

Powertrain test rig	Experimental fuel	Modelling and optimization framework	Optimized input factors	Output responses	Desirability value	Reference
1 cylinder, 3.5 kW, 17.5:1 CR, 1500 rpm, 21°CA bTDC, Kirloskar model, vertical type, DI diesel engine	Diesel + bael biodiesel + dimethyl carbonate (DMC) blends	RSM with DFA	50% engine load, 10% bael biodiesel blend, and 3.59% DMC additive	BTE (24.05%), BSFC (0.4419 kg/kW hr), CO (0.203%), HC (113 ppm), NO_x_ (316 ppm), and smoke opacity (14.43%)	0.7941	^23^
One cylinder, 4 S, 12:1–18:1 CR, 3.5 kW BP, VCR engine	Aegle marmelos (AM) pyrolysis oil/diesel/Tert-butyl hydroxyl quinone antioxidant (TBHQ) blends	RSM with DFA	20% AM pyrolysis oil, 100% engine load, and 17.5:1 CR	BTE (22.01%), BSFC (0.33 kg/kWh), CO (0.67%), HC (244 ppm), CO_2_ (8.33%), and NO_x_ (351 ppm)	0.917	^24^
Single cylinder, 4 S, 3.5 kW BP, 1500 rpm, 12:1–18:1 CR range operated diesel engine	Jatropha curcas shell bio-oil-diesel blends	RSM-CCD with DFA	18:1 CR, 6.67 kg load, and 12.2% fuel blend	BTE (17.45%), BSFC (0.46 kg/kWh), CO (0.1%), UHC (56.75 ppm), and CO_2_ (5.06%)	0.786	^25^
1 cylinder, 4 S, vertical type, 12 kg load, 16:1–18:1 CR, 1500 rpm, computerized VCR engine	Argemone mexicana biodiesel (AMB) -diesel blends	RSM-FFD with DFA	9.8 kg engine load, 18:1 CR, 20% AMB blend	BTE (26.77%), BSFC 0.284 (kg/kWh), CO (0.0059%), HC (114.84 ppm), and NO_x_ (906 ppm)	0.9701	^26^
1 cylinder, 4 S, 19:1 CR, 3.75 kW BP, 1500 rpm, 23°CA bTDC, Kirloskar TV1 model, VCR engine	Waste cooking soybean oil (WSCO) biodiesel blends	RSM with DFA	100% engine load, 25% WSCO biodiesel blend, and 15% EGR	BTE (30.56%), BSFC (0.271 kg/kWh), CO (0.069%), HC (19.98 ppm), smoke (25.67%), and NO_x_ (66.08 ppm)	0.9282	^27^
Single cylinder, evaporation cooling, 17.6:1 CR, 18 kW BP, 2200 rpm, ZS1115NM model diesel engine	Waste cooking oil (WCO) biodiesel blends	RSM-CCD with DFA	2.67 kW engine load, 100% pure diesel	BTE (17.11%), NO_x_ (658.92 ppm), and CO_2_ (1.94%)	0.6574	^28^
1 cylinder, 4 S, water cooled, 17.5:1 CR, 3.5 kW BP, 1500 rpm, 23°CA bTDC, 200 bar injection pressure, Kirloskar brand CI engine	Mahua biodiesel (MB)/diesel + CuO nanoparticle	RSM-CCD with DFA	80% engine load, 20% MB + 60 ppm CuO	BTE (33.32%), BSFC (0.2771 kg/kWh), Peak CP (71.13 bar), HRR (61.91 J/^o^CA), CO (0.0689%), HC (50.02 ppm), smoke (31.46%), and NO_x_ (807.98 ppm)	0.9740	^29^
One cylinder, 4 S, 1500 rpm constant speed, 5.2 kW BP, 17.5:1 CR operated Kirloskar TV1 model engine	Tectona grandis biodiesel (TB) + Elaeocarpus ganitrus additive (R)	RSM with DFA	20% TB + 5% R, 68 kg load, and 17.5:1 CR	BTE (31.52%), BSFC (0.31 kg/kWh), EGT (250 °C), CO (0.158%), CO_2_ (4%), HC (74.31 ppm), NO_x_ (800 ppm), and smoke opacity (50%)	0.9491	^30^

## Materials and methods

### Lemon cultivation and its waste materials use

**Fig. 1:**
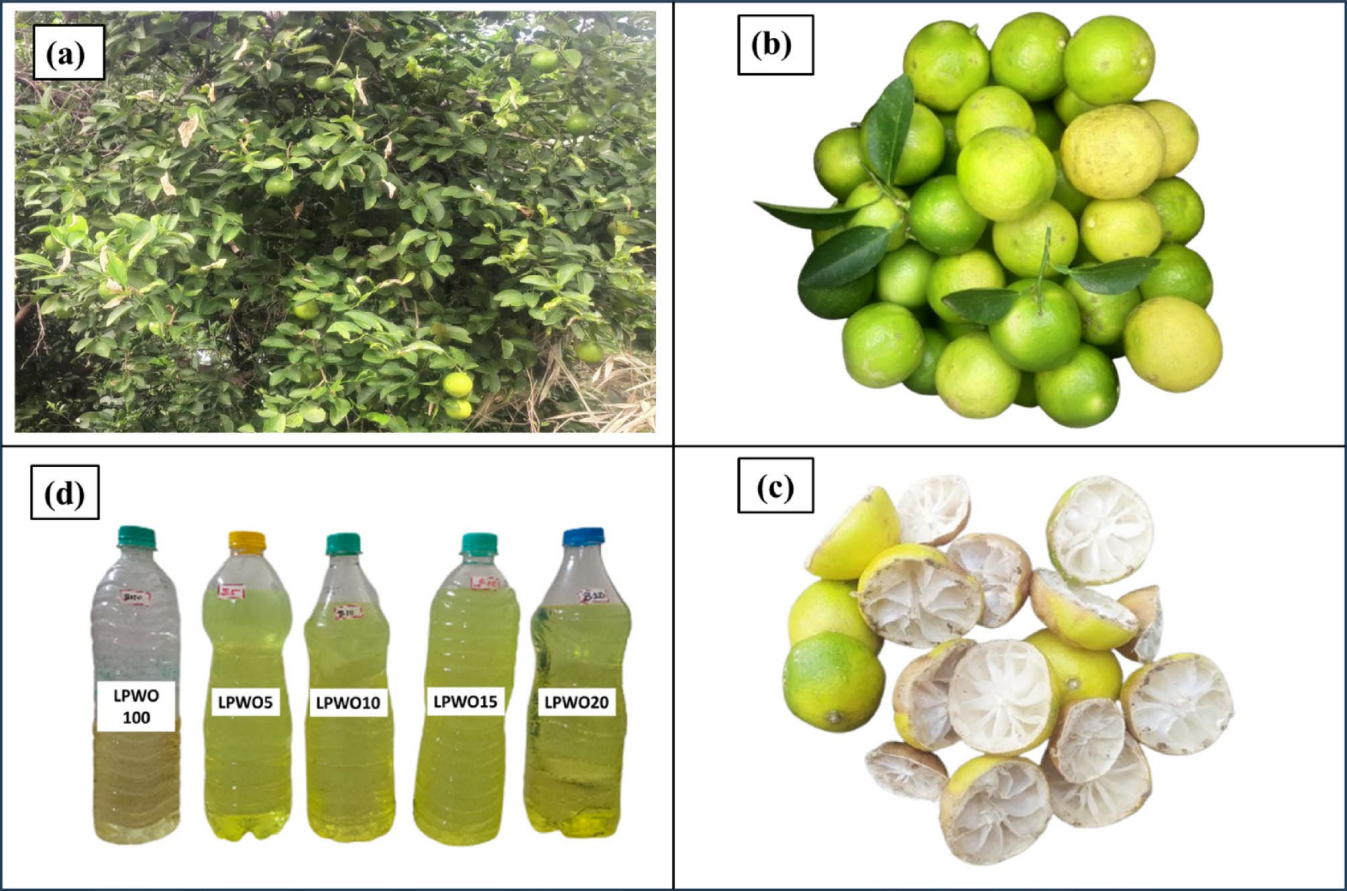
(**a**) Lemon tree (*Citrus limon*), (**b**) Fresh lemon fruit, (**c**) Lemon peel waste, (**d**) Lemon peel waste oil and its diesel blends (The corresponding author **(R. M)** personally captured all the images).

The lemon tree (*Citrus limon*) is a widely cultivated fruit-bearing tree in India, valued for its adaptability, economic importance, and various uses. It belongs to the Rutaceae family and thrives in tropical and subtropical climates, making the Indian environment ideal for its growth. The tree is small to medium-sized, evergreen, and known for its fragrant flowers and vitamin C-rich fruits, as presented in Fig. 1. Major lemon-growing states in India include Andhra Pradesh, Gujarat, Maharashtra, Karnataka, Madhya Pradesh, and Tamil Nadu^31^. These regions offer the ideal warm climate and well-drained soil for healthy growth. Lemon trees can bear fruit year-round, with peak summer and rainy seasons. Economically, lemon cultivation provides a steady income for farmers due to constant demand in households, food industries, and traditional medicine. With rising demand for organic produce, lemon farming increasingly uses sustainable practices, adding to its environmental and economic value.

Traditionally, lemon peels were considered waste and often discarded or used as compost. However, with growing awareness of sustainability and waste management, lemon peel waste is now being repurposed in innovative and environmentally friendly ways. One popular method involves converting lemon peels into natural cleaning agents. When fermented with jaggery and water, the peels produce a bio-enzyme solution that is a practical, chemical-free cleaner for household use^32^. Besides cleaning products, lemon peels also have applications in the culinary world. Rich in essential oils and nutrients, lemon zest enhances the flavour of various dishes and is used in baking, garnishing, and cooking^33^. Using lemon peel waste for biofuel production helps in energy recovery and supports circular economy practices by converting agricultural waste into valuable green fuels^34^. With proper technological integration and scale-up, lemon peels can play a significant role in India’s renewable energy sector.

### Lemon peel waste oil preparation

**Fig. 2:**
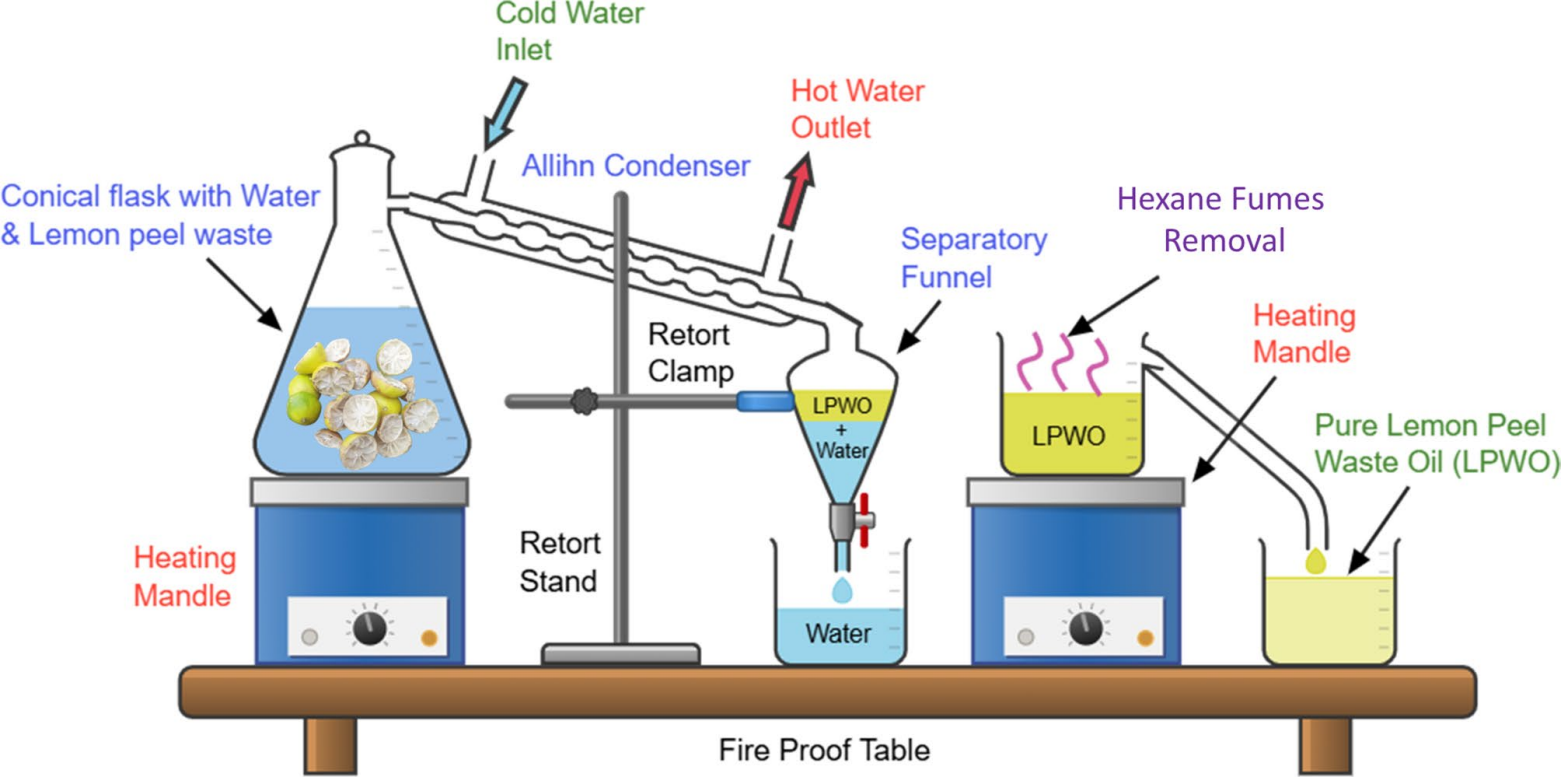
Steps carried out for the production of lemon peel waste oil (LPWO) (The corresponding author **(R. M)** personally framed the process layout through https://chemix.org).

The lemon oil is extracted from waste lemon peels by the steam distillation process, and the extraction process is indicated in Fig. 2. The heating mantle is used to heat at 100 °C the mixture of mineral water and the marinated lemon peels. To prevent leaks and maintain the pressure of the steam leaving the conical flask, the nozzle is equipped with an Allihn condenser. The condensation system consists of an inlet port that passes the cold water to cool the lemon vapour, and hot water is removed using an outlet port. In this condensation process, the liquid mixture of mineral water and lemon peel oil is collected in the separatory funnel. Separation is based on the difference in density; since water is heavier than lemon peel waste oil (LPWO), it settles at the bottom of the separatory funnel, allowing the LPWO (along with its impurities) to be easily extracted from the top. The collected top layer LPWO is mixed with hexane, and the mixture is heated at around 68 °C in a heating mantle, causing the hexane fumes and other volatile substances to evaporate. Finally, the remaining solid impurities are removed using a suitable filter paper, which gives pure LPWO and is collected in the beaker. In this study, experimental blends were prepared by mixing pure LPWO with diesel fuel in volume ratios of 5%, 10%, 15%, and 20%. These blends were designated as LPWO5, LPWO10, LPWO15, and LPWO20, corresponding to the percentage of LPWO in each mixture. Also, their various fuel characteristics are listed in Table 2.

**Table 2:** Various fuel characteristics of diesel, pure LPWO and lpwo/diesel blends.

Fuel Properties	Diesel	LPWO5	LPWO10	LPWO15	LPWO20	LPWO 100	ASTM methods	Equipment utilized
Kinematic viscosity (mm^2^/s) @ 40 °C	2.93	2.84	2.76	2.70	2.59	1.22	ASTM D445	Redwood viscometer
Density (kg/m^3^) @ 15 °C	835	837	838	840	841	851	ASTM D1298	Glass hydrometer
Lower heating value (MJ/kg)	42.5	42.44	42.40	42.35	42.31	41.52	ASTM D240	Bomb calorimeter
Flash point (°C)	67	65	64	62	61	51	ASTM D93	Closed cup Pensky-Martens apparatus
Fire point (°C)	81	79	78	76	75	62	ASTM D93	
Cetane number	52	50	48	47	45	15	ASTM D976	Traditional laboratory method

## Design and experimental protocol for the VCR engine

### VCR engine design

The engine used in the current investigation is a one-cylinder, four-cycle, liquid-cooled, CI (Kirloskar manufacture) engine that delivers a maximum power of 5.2 kW at a rotational speed of 1500 rpm. An eddy current dynamometer is connected to the VCR engine to function under various loading conditions of 0 to 5.2 kW with 1.3 kW variations for six test fuels (Diesel, LPWO5, LPWO10, LPWO15, LPWO20, and LPWO100). Table 3 contains the test VCR engine’s technical specifications.

**Table 3:** Technical details of the VCR engine.

Engine make and variant	Kirloskar diesel engine and TV-1 (Computerized)
Cylinder bore (D)/stroke (S)	87.50/110 mm
CR variations	16:1, 17:1 and 18:1
Engine-maximum power/speed	5.2 kW (7 hp)/1500 rpm
Swept capacity of the engine	661.45 cm^3^
Fuel delivery pressure and injection schedule	200 bar & 23 degrees CA before TDC
Con-rod (*l*)/Dynamo (R_m_) arm length	234/185 mm
Design of engine piston bowl	Hemispherical shape
Quantity of injector holes (n)/size (d)	3 nos./0.3 mm
Dynamometer description	Make - Techno Mech, Model - TMEC 10, Type - Eddy current, Speed Range − 1500 to 6000 RPM, Power − 7.5 kW (Maximum)
Pressure sensor	Make - PCB Piezotronics, INC. Model - M111A22, Type - Piezoelectric pressure sensor, Measuring Range − 5000 to 15,000 psi, with low noise cable, Sensitivity − 1 mV/psi
Crank angle position sensor	Resolution − 1 deg, Speed − 5500 rpm with TDC pulse
Data acquisition system	Make - National Instruments Corp. Model - NI USB-6210, Input Range - ±0.2 V to ± 10, ADC resolution − 16-bit, Sample Rate − 250 kS/s
Temperature transmitter	Type two-wire, Input RTD PT100, Range − 0 to 100 °C, I/P Thermocouple (Type K), Range – 0 to 1200 °C, O/P 4 to 20 mA
Load sensor	Load cell, Type - Strain gauge, Range – 0 to 50 kg
Fuel flow transmitter	Model - DP transmitter, Range – 0 to 500 mm WC
Airflow transmitter	Model - Pressure transmitter, Range - (-) 250 mm WC
Fuel supply injection unit	MICO in-line model with a mechanical governor

**Fig. 3:**
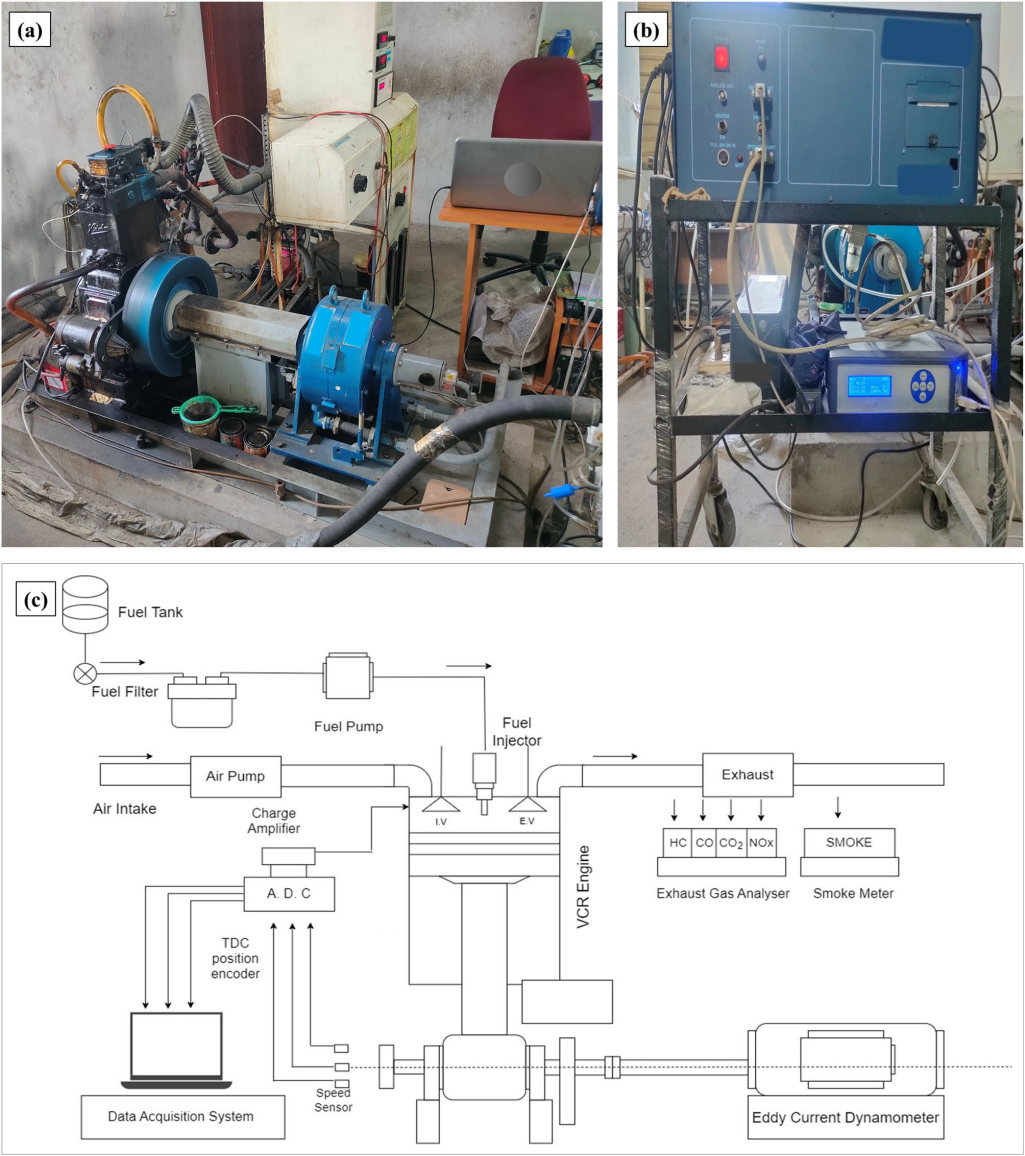
**(a)** Pictorial representation of VCR engine, and **(b)** Exhaust anlyzer/Smoke meter setup. **(c)** Complete experimental layout of VCR engine (The corresponding author **(R. M)** personally captured the images and framed the layout).

For this experiment, the compression ratio of the CI engine is varied by utilizing Allen bolts to pivot the cylinder head; all test values for measurements showed compression ratios varying from 16:1 to 18:1. The CR adjuster changed the clearance volume by changing the cylinder head. The variation in the compression ratio is displayed by the CR indicator. In this engine, the open electronic control unit (ECU) has been used to convert the traditional injection into electronic injection along with the appropriate sensors and actuators. The ECU employed in this investigation may dynamically alter the direct injection time duration in response to engine speed and load circumstances. A data acquisition setup was utilized to obtain combustion data from the engine; a crankshaft angular position encoder and a pressure sensor were mounted on the test VCR engine. Also, a K-type thermocouple connected to the engine exhaust is used to measure EGT. Exhaust emissions of CO, HC, NO_x_ and CO_2_ are analyzed using an AVL DI gas 444 N five-gas analyzer. A smoke meter of the AVL 437 C type is used to monitor smoke emission. Comprehensive technical specifications of the exhaust emission analyzer and smoke meter instruments are listed in Table 4. The design of the VCR engine, exhaust emission analyzer/smoke meter used and the experimental layout are illustrated in Fig. 3**(a)**,** (b)** and **(c).**

**Table 4:** In-depth engineering specifications of the emission analyzer and smoke measurement device.

Exhaust emission analyzer specifications			
Parameters	Measuring range (volume-based)	Resolution (volume-based)	Accuracy (volume-based)
CO emission	0 to 15%	0.01%	0 to 10%
HC emission	0 to 30,000 ppm	≤ 2000: 1 ppm	0 to 4000 ppm: ±8 ppm
NO_x_ emission	0 to 5000 ppm	1 ppm	± 5 ppm
Smoke meter specifications			
Parameters	Measurement span	Sensitivity	Precision
Smoke	0 to 100%	0.1%	± 1%
Absorption (K value)	0 to 99.99 m^−1^	0.01 m^−1^	± 0.1 m^−1^
Engine speed (rpm)	400 to 6000 1/min.	± 1	± 10 (1/min)
Oil temperature	0 to 150 °C	± 1 °C	± 2 °C

### Experimental test protocol

The performance of the VCR engine was assessed at varying load conditions from 0 to 5.2 kW with 1.3 kW intervals, under three different compression ratios: 16:1, 17:1, and 18:1. Prior to initiating the experimental runs, the thermal condition of the VCR test engine, along with the flow of coolant and lubricating oil, as well as the fuel level, are thoroughly inspected. The flow of coolant and lubricating oil, along with the load accuracy of the eddy current brake dynamometer, was checked and aligned. To begin, the VCR setup was operated for 30 min to reach stable operating conditions. After the test, the VCR engine attains steady-state operation, and various fuel blends, Diesel, LPWO5, LPWO10, LPWO15, LPWO20, and LPWO100, are evaluated under different load conditions. Each fuel blend test was conducted three times to ensure measurement precision, and the average of the results was used for graph plotting to maintain consistency and reliability. All experimental graphs are plotted using OriginPro 2022 software version 9.9.0.225 (www.originlab.com). Table 5 presents the experimental matrix for the VCR engine test fuels, outlining the blending ratios of diesel and LPWO.

**Table 5:**
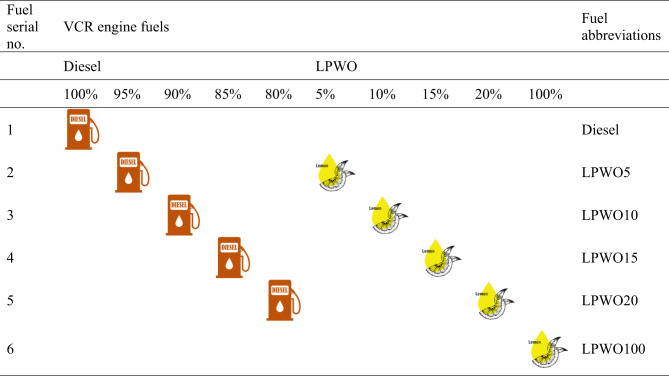
Fuel test matrix of diesel/lpwo blends for varied CR values.

### Uncertainty

The percentage uncertainties were calculated for all measured constraints, including VCR engine load (BP) and speed (RPM), performance variables (BSFC, BTE, and EGT), tailpipe emissions (unburnt HC, CO, NO_x_, and CO_2_) and smoke. The total uncertainty of the experimental measurements is calculated using Eq. (1), applying the square root method to assess the combined uncertainties of all parameters^35^. This approach ensures a comprehensive evaluation of measurement accuracy and reliability.

1 $$\:=\sqrt{\begin{array}{c}{\left({U}_{BP}\right)}^{2}+{\left({U}_{RPM}\right)}^{2}+{\left({U}_{BSFC}\right)}^{2}+{\left({U}_{BTE}\right)}^{2}+{\left({U}_{EGT}\right)}^{2}+{\left({U}_{HC}\right)}^{2}+{\left({U}_{CO}\right)}^{2}+\\\:{\left({U}_{{NO}_{x}}\right)}^{2}+{\left({U}_{{CO}_{2}}\right)}^{2}+{\left({U}_{Smoke}\right)}^{2}\\\:\end{array}}$$

$$\:=\sqrt{{\left(1\right)}^{2}+{\left(1\right)}^{2}+{\left(1.5\right)}^{2}+{\left(2\right)}^{2}+{{\left(0.1\right)}^{2}+\left(1.2\right)}^{2}{+\left(0.7\right)}^{2}+{\left(1\right)}^{2}+{\left(0.1\right)}^{2}+\:{\left(1.5\right)}^{2}}$$

$$\:=\:\pm\:\:3.67\%$$

## Response surface methodology

### Design of experiment

Response surface methodology (RSM) is a suite of statistical techniques for modelling and analyzing problems in which several variables influence the outcomes. In practice, it is critical to choose sample points in a manner that allows for the development of a credible model with the fewest number of trials^36^. These points are chosen using an experimental design. The quadratic response surface models were created by including the impacts of response variables obtained from a central composite design (CCD) matrix using Design-Expert 13.0.15 software (https://www.statease.com/software/design-expert/). In the present study, factors such as compression ratio (CR), brake power (BP), and LPWO blend ratio (B) reaction were regarded as input variables that could be analyzed to determine the effect of operational and emission metrics in CI engine applications. Three distinct variables of the VCR engine, along with their respective operational phases in the CCD model, are listed in Table 6.

**Table 6:** Three different variables of the VCR engine and their corresponding phases.

VCR engine variables	Symbol	Phases		
		−1	0	+ 1
Compression ratio	CR	16:1	17:1	18:1
Brake power (kW)	BP	0	3.6	5.2
LPWO blend ratio (%)	B	0	10	20

Based on Table 6 inputs, the CCD matrix is generated for 15 VCR experimental runs with varied factor-level combinations. Three centre points were inserted in each block to quantify experimental error and increase CCD model estimation precision. Table 7 summarizes the VCR experimental results using the CCD-based RSM. The obtained data was analyzed to create a predictive model and determine the most important elements influencing VCR engine output responses.

**Table 7:** VCR experimental variables and their corresponding outcomes using the RSM-CCD model.

Std order	Run order	CR	BP (kW)	B (%)	BTE (%)	BSFC (kg/kWh)	EGT (^o^C)	HC (ppm)	CO (%)	NO_x_ (ppm)	Smoke (%)	CO_2_ (%)
3	1	17	5.2	0	32.36	0.25	371.95	0.085	41	1846	67.8	9.21
13	2	17	2.6	10	27.47	0.31	231.83	0.034	43	1155	23.8	5.22
5	3	16	2.6	0	18.73	0.29	239.89	0.014	29	953	17.5	4.1
14	4	17	2.6	10	27.47	0.31	231.83	0.034	43	1155	23.8	5.22
11	5	18	0	10	1.3	4.24	21.23	0.048	36	93	1.1	1.9
7	6	18	2.6	0	21.87	0.33	232.88	0.011	20	1134	15.3	5.23
8	7	18	2.6	20	26.99	0.34	232.57	0.039	38	1132	27.4	5.938
6	8	16	2.6	20	25.34	0.323	239.66	0.053	41	943	31.9	4.2
10	9	16	5.2	10	30.98	0.257	381.6	0.077	70	1952	72.6	8.34
4	10	17	5.2	20	31.94	0.27	381.41	0.14	84	1953	67.8	9.97
15	11	17	2.6	10	27.47	0.31	231.83	0.034	43	1155	23.8	5.22
2	12	17	0	20	0.89	3.25	14.29	0.084	35	40	5.6	1.38
12	13	18	5.2	10	33.765	0.263	381.6	0.059	65	2148	56.4	10.12
9	14	16	0	10	1.23	5.04	9.37	0.072	39	73	2.4	1.36
1	15	17	0	0	0.91	3.89	18.23	0.001	5	149	5.8	1.31

### DFA optimization

The regression equations obtained through RSM were further optimized with DFA, an extensively adopted technique for handling multi-objective optimization problems. Each response is converted into a dimensionless individual desirability value (*d*_*i*​_) ranging from 0 (least desirable) to 1 (most desirable). A value close to 1 indicates the response meets the target or goal, while a value near 0 signifies it falls outside acceptable limits^37^. Depending on the problem, the individual response objective can be defined to achieve a maximum, minimum, or specific target value, fall within a range, or match a specific value. The overall desirability (*d*_*o*_) values are calculated as the weighted geometric mean of the specific desirability (*d*_*i*_*)* counts for all output metrics, as expressed in Eq. (2).

2 $$\:{d}_{o}=\sqrt[n]{{d}_{1}\times\:{d}_{2}\times\:{d}_{3}\dots\:{d}_{n}}$$

Here, n is the no. of output metrics. The *d*_*o*_ score of the linear model with multiple independent variables is 0.8997, indicating proximity to the ideal value of 1. This signifies that the optimized outcomes are highly suitable for the chosen VCR engine test setup.

## Results and discussion

### Characterization study on LPWO

#### FTIR report

**Fig. 4:**
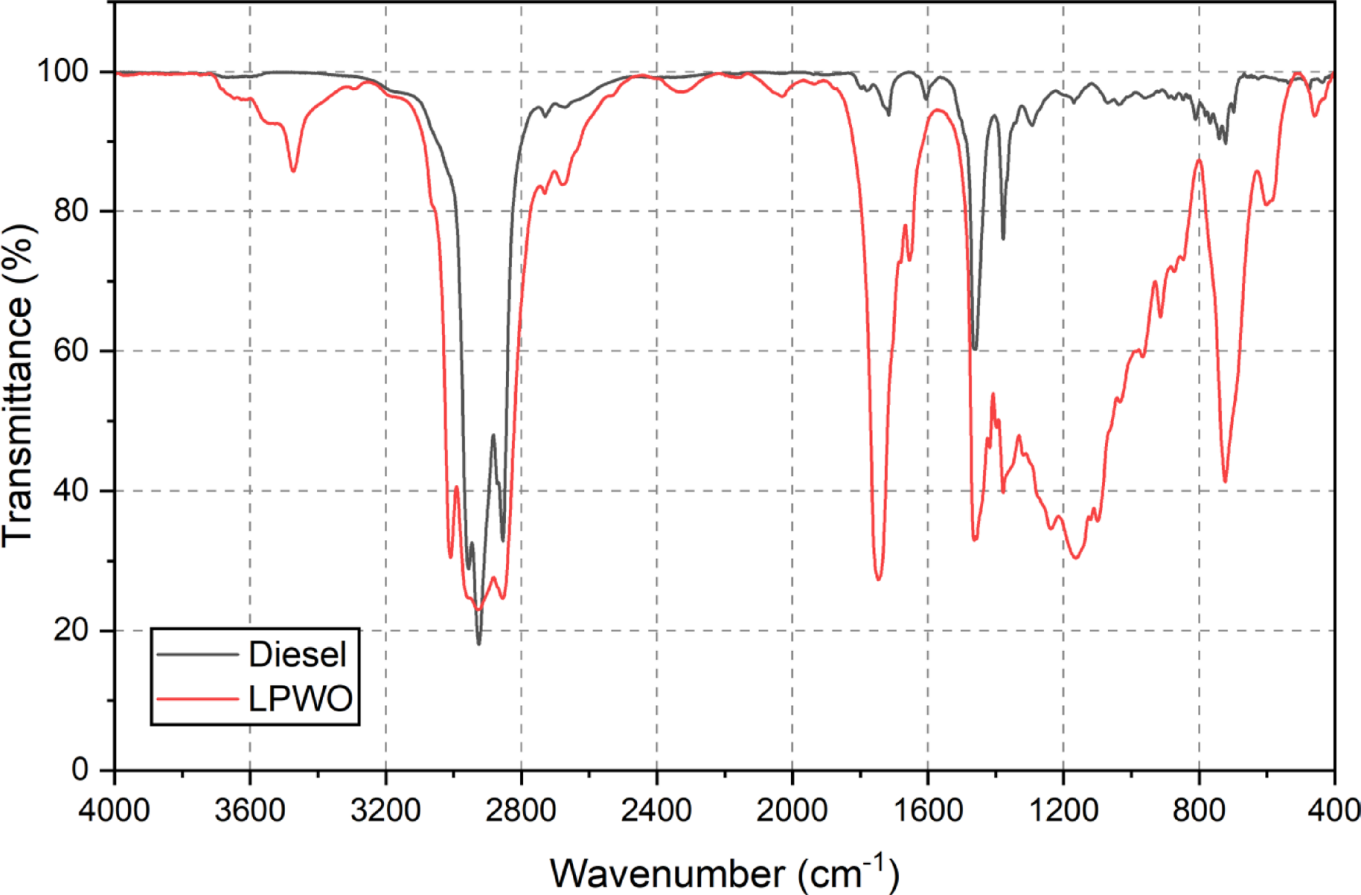
FTIR profiles for standard diesel and LPWO.

**Fig. 5:**
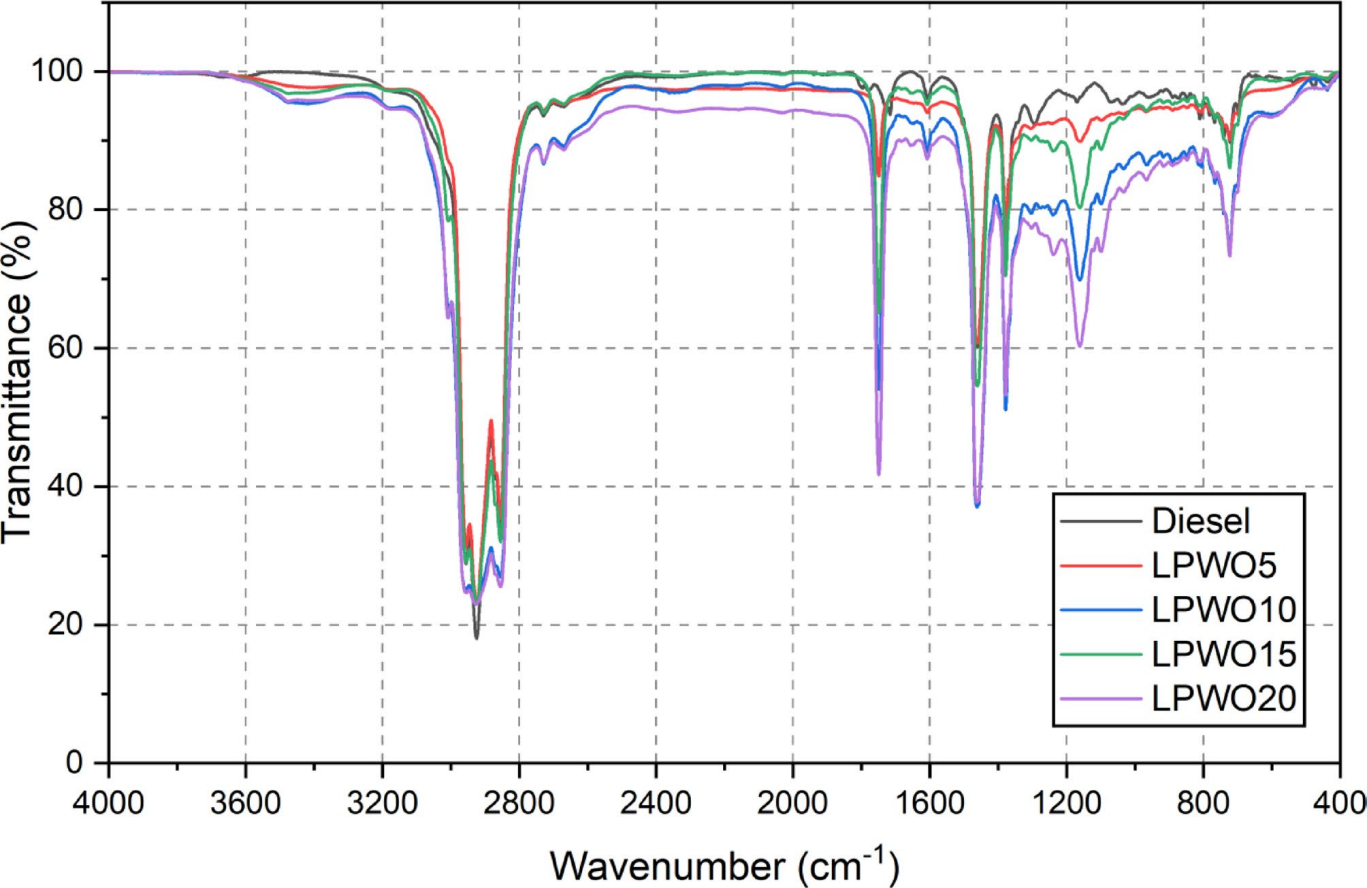
FTIR profiles for standard diesel and LPWO fuel blends.

Fourier-transform infrared spectroscopy (FTIR) was employed to obtain the absorption spectra of the samples, utilizing the transmission mode within the wavenumber range of 4000 cm^−1^ to 400 cm^−1^, as proved in Figs. 4 and 5. The FTIR analysis of LPWO revealed the presence of various functional groups^38^with the results summarized in Table 8. A prominent presence of alkanes and aliphatic compounds characteristic of conventional diesel was observed in LPWO. Additionally, LPWO exhibited a strong presence of mono-alkyl esters, aldehydes, and ketones, which were not prominent in diesel. A mild presence of aromatic compounds, phenols, and alcohols was also detected. Furthermore, LPWO contained significant amounts of acids, esters, alkenes, and tertiary alcohols^39^. When comparing different fuel blends, LPWO5 (5% LPWO and 95% diesel) closely resembled the FTIR profile of pure diesel. However, as the LPWO content increased to 20% in diesel, there was a marked rise in the intensity of peaks associated with aromatics, acids, esters, and alcohols, indicating enhanced contribution of oxygenated and unsaturated compounds from the LPWO.

**Table 8:** Functional moieties in LPWO through FTIR profiles.

Wavenumber (cm^−1^)	Functional moieties and Nature of vibration	Degree of absorption	Chemical grouping	Reference
3485.96	O-H and Stretching mode	Low	Alcohols and Phenolic compounds	^40^
3014.21	=C-H and Asymmetric stretching	High	Saturated hydrocarbons	
2836.44	C-H and Stretching motion	High	Aliphatic chains	^41^
1763.68	C = O and Stretching motion	High	Esters, Aldehydes, and Ketones	^42^
1477.23	C = C and Stretching mode	Moderate	Unsaturated hydrocarbons, Aromatic rings	
1389.3	C-OH and Bending mode	Moderate	Secondary and Tertiary alcohols	^43^
1183.47	C-O and Stretching vibration	High	Esters and Organic acids	
736.76	C-H and Bending motion	Moderate	Aromatic compounds	^44^

#### GC-MS analysis

The chemical composition of LPWO is further examined using a gas chromatography-mass spectrometer (GC-MS)^45^. It was carried out in a Shimadzu GC-MS QP2010 Plus analyzer. GC and MS both analyse gaseous compounds at high temperatures, but differ in pressure requirements. GC operates at atmospheric pressure, while MS requires a high vacuum (10^−3^ to 10^−4^ Pa). An interface and vacuum system are essential to bridge this pressure gap and enable effective coupling of the two techniques. The main constituents of LPWO are presented in Table 9. The dominant compound is bis-(2-ethylhexyl) terephthalate, accounting for 31.67% of the peak area, indicating a strong presence of esters and, consequently, a significant oxygen content in LPWO^46^. The second major component is butyl 2,4-dimethyl-2-nitropent-4-enoate, with a peak area of 9.54%, suggesting the presence of nitrogen-containing compounds in the LPWO.

**Table 9:**
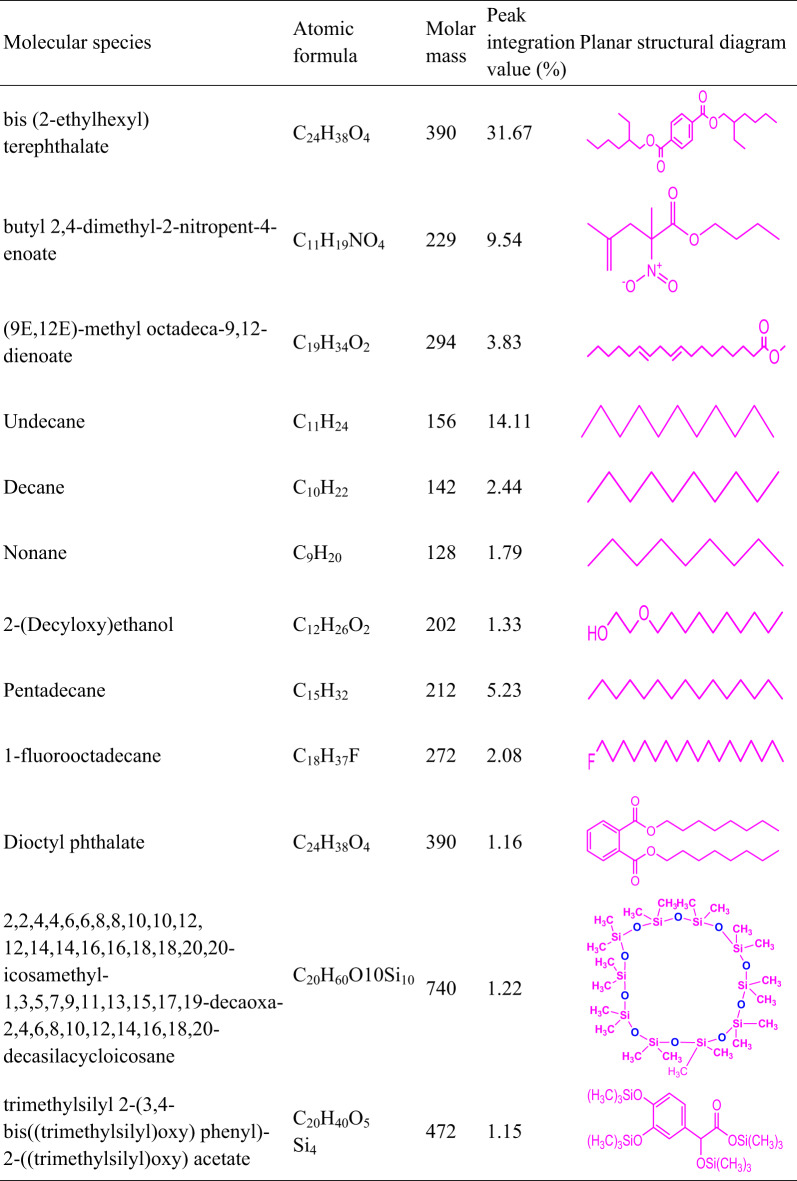
Substantial proportion of identified chemical constituents in LPWO.

#### TG/dTG analysis

**Fig. 6:**
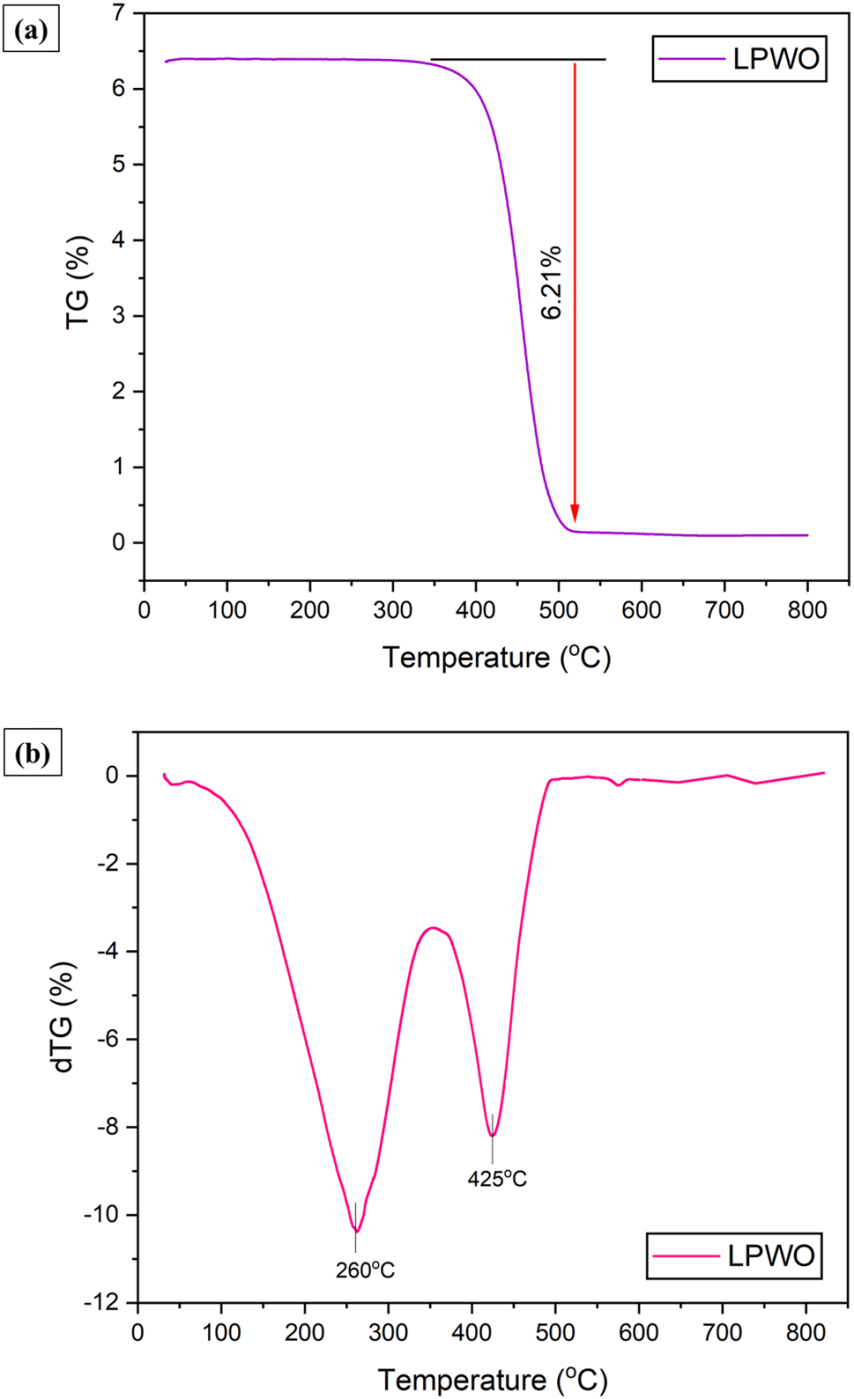
**(a)** TG curve for the LPWO. **(b)** dTG curve for the LPWO.

The thermogravimetric (TG) analysis of LPWO was conducted to assess its thermal stability and decomposition characteristics by heating the sample (LPWO) from ambient temperature to 800 °C, as shown in Fig. 6**(a).** The TG analysis was conducted under dry air and nitrogen (N₂) environments, with the temperature ramped consistently at 8 °C per minute and a weighing precision of 0.1 mg^40^. The TG analysis curve shows a single major weight loss event occurring between approximately 360 °C and 512 °C, corresponding to a total mass loss of 6.21%. The negligible weight loss observed up to 360 °C indicates that LPWO exhibits high thermal stability within this range. The sharp decline in mass beyond 360 °C suggests rapid decomposition or volatilization of organic components such as long-chain fatty acids and esters. After 512 °C, the curve stabilizes, indicating the completion of thermal degradation^41^. Overall, the low total mass loss and high thermal stability suggest that LPWO contains predominantly thermally stable compounds, making it a potentially valuable candidate for applications like biofuel production.

The derivative thermogravimetry (dTG) of LPWO was conducted to investigate its thermal decomposition behaviour and stability over a temperature range of 25 °C to 800°C^47^, as presented in Fig. 6. **(b)**. The corresponding DTG curve reveals two distinct peaks, indicating that the decomposition occurs in two overlapping stages. The first major peak, observed around 260 °C, corresponds to the initial degradation of lighter volatile compounds such as short-chain fatty acids or residual moisture-organic complexes. The second, more prominent peak around 425 °C represents the primary thermal degradation of more stable and heavier organic constituents, likely long-chain triglycerides and esters. After this stage, the DTG curve levels off, confirming the completion of decomposition. These findings demonstrate that LPWO possesses good thermal stability and decomposes in a stepwise manner, making it a suitable candidate for thermochemical conversion processes such as biodiesel production, pyrolysis, or catalytic upgrading in CI engine applications^48^.

### Analysis of variance (ANOVA)

By applying a multiple regression model, the dataset was fitted to a quadratic equation, resulting in a data-driven model that describes the relationship between the output metrics and the influencing input factors^49^. ANOVA was performed using Design-Expert version 13.0.15 (https://www.statease.com/software/design-expert/) to evaluate the statistical relevance of each term in the developed equations, confirming the adequacy of the model fit and the desirability of the adjustment metrics. Terms were considered significant if they exhibited an F-value within the 95% confidence interval and a p-value less than or equal to 0.05. Optimal values of the selected variables were determined by analyzing the response surface (RS) plots and solving the regression model^30^. The developed quadratic equation was represented through 3D RS graphs to illustrate both the individual and interactive effects of the independent variables on the response outcomes.

The adequacy of the RSM model using CCD was evaluated through ANOVA based on the least squares method. ANOVA is an effective statistical tool that breaks down the total variability in the model data into separate sources, enabling a clearer understanding of each factor’s individual contribution^37^. Table 10 presents the ANOVA outcomes for both performance parameters and exhaust emission behaviour of the VCR engine. The analysis focuses primarily on two key statistical indicators: the probability value (p-value) and Fisher’s test value (F-value). These metrics help determine the significance of the developed quadratic regression models. A p-value less than 0.05 indicates that the model is statistically significant, suggesting that changes in input variables lead to meaningful differences in the responses, thereby rejecting the null hypothesis. Conversely, a p-value greater than 0.05 implies model insignificance^38^. As shown in Table 10, all response models exhibit p-values below 0.05, confirming the statistical significance of the quadratic models for both performance and emission outputs. Specifically, for LPWO optimization, the model is found to be significant for BTE (*p* < 0.05), highlighting its effectiveness in enhancing BTE. However, the models for BSFC and EGT under LPWO conditions yield p-values above 0.05, indicating that these responses are not significantly influenced. Regarding emission parameters, controllable inputs linked to the CR, BP, and B show p-values below 0.05, confirming their strong influence on emissions. The adequacy of the model fit was evaluated through regression metrics such as the coefficient of determination (R²), adjusted R², and predicted R². Among the responses, EGT exhibited the highest R² value (0.9728), followed by BSFC (0.9705) and BTE (0.9489), reflecting excellent model accuracy across all outputs. For emission parameters, the R² values are 0.9674 (HC), 0.9947 (CO), 0.9877 (NO_x_), 0.9827 (smoke), and 0.9931 (CO₂). These high R² values, all close to unity, demonstrate that the RSM-CCD model effectively captures the relationships between the VCR engine’s input conditions and output responses.

**Table 10:** Statistical evaluation of VCR engine performance and exhaust emission metrics models through ANOVA.

Metrics	BTE		BSFC		EGT		HC		CO		NO_x_		Smoke		CO_2_	
Source	F value	P value	F value	P value	F value	P value	F value	P value	F value	P value	F value	P value	F value	P value	F value	P value
Model	134.05	< 0.0001*	9.59	< 0.0001*	258.35	< 0.0001*	47.24	< 0.0001*	16.43	< 0.0001*	578.95	< 0.0001*	411.07	< 0.0001*	1043.43	< 0.0001*
Compression ratio (CR)	4.11	0.0468*	0.2070	0.6507	0.3249	0.5707	8.93	0.0040*	7.31	0.0087*	32.15	< 0.0001*	55.22	< 0.0001*	305.20	< 0.0001*
Brake power (BP)	1040.10	< 0.0001*	16.44	< 0.0001*	2256.25	< 0.0001*	237.48	< 0.0001*	11.69	0.0011*	5144.20	< 0.0001*	3371.49	< 0.0001*	8985.66	< 0.0001*
LPWO blend ratio (B)	13.68	0.0004*	0.5375	0.0043*	0.1934	0.6616	94.99	< 0.0001*	59.55	< 0.0001*	2.25	0.1387	64.66	< 0.0001*	17.97	< 0.0001*
CR*BP	1.39	0.2430	0.0131	0.9094	0.0021	0.9639	1.44	0.2348	0.1859	0.6678	9.82	0.0026*	13.41	0.0005*	44.27	< 0.0001*
CR*B	0.1378	0.7117	0.0245	0.0061*	0.0010	0.9746	1.33	0.2536	1.10	0.0029*	0.2006	0.6557	0.0113	0.9156	14.40	0.0003*
BP*B	5.19	0.0260*	0.0173	0.8959	0.0881	0.0034*	5.76	0.0192*	0.9093	0.3438	2.34	0.1309	2.28	0.1362	4.30	0.0421*
CR²	0.0384	0.8453	0.0000	0.9969	0.0251	0.8745	0.0062	0.9376	0.2676	0.6067	1.28	0.2628	0.3855	0.5368	0.0156	0.9009
BP²	121.98	< 0.0001*	69.11	< 0.0001*	68.22	< 0.0001*	39.49	< 0.0001*	66.68	< 0.0001*	4.13	0.0462*	185.45	< 0.0001*	16.91	0.0001*
B²	19.83	< 0.0001*	0.0007	0.9784	0.0146	0.9041	35.78	< 0.0001*	0.2208	0.6400	14.22	0.0004*	6.73	0.0117*	2.13	0.1490
Residual	589.19		0.7048		33246.73		2785.33		0.0210		4.704E + 05		663.70		4.17	
Cor Total	11525.12		1.64		1.223E + 06		21005.69		0.0688		3.818E + 07		38439.56		606.91	
R^2^	0.9489		0.9705		0.9728		0.9674		0.9947		0.9877		0.9827		0.9931	
Adjusted R^2^	0.9418		0.9111		0.9690		0.9490		0.9524		0.9860		0.9803		0.9922	
Predicted R^2^	0.9286		0.9461		0.9649		0.9081		0.9516		0.9829		0.9754		0.9906	

*Significant factor

### VCR engine performance characteristics

#### Brake thermal efficiency (BTE)

**Fig. 7:**
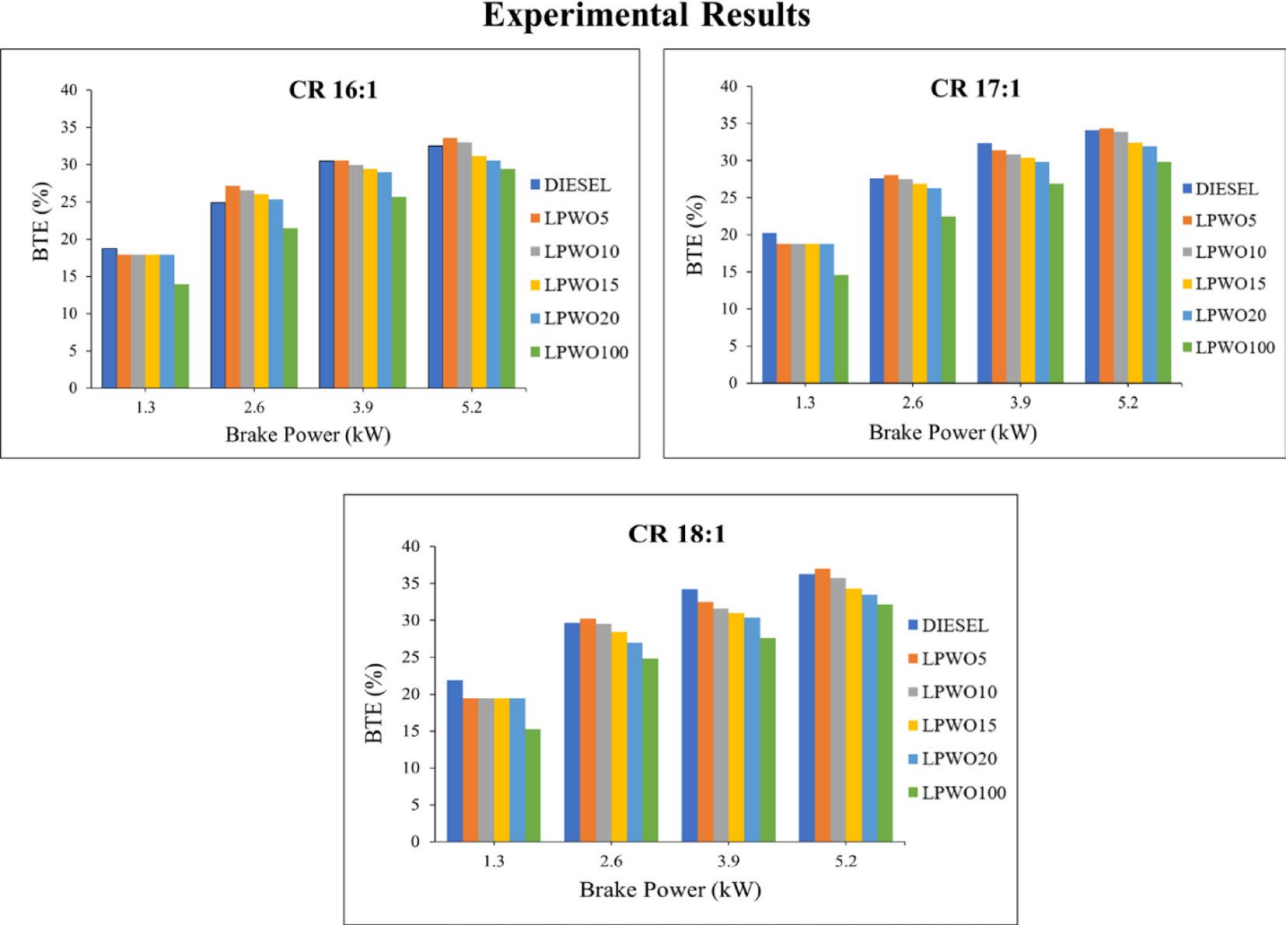
Experimental results of BTE with respect to BP at CRs of 16:1, 17:1, and 18:1 for diesel and different LPWO blend ratios.

A measure of an engine’s ability to convert fuel energy into useful mechanical energy is called BTE. The more effectively a system converts fuel energy into usable work, the greater its BTE^21^. Figure 7 depicts the experimental outcomes of BTE with BP output for compression ratios 16:1, 17:1, and 18:1. As CR and BP increase, BTE for LPWO5, LPWO10, LPWO15, and LPWO20 is more than that of neat diesel, and LPWO100 shows BTE more than diesel but less than the blends. As a result, it can be said that LPWO combined with diesel demonstrates enhanced BTE for all engine load and CR variations. LPWO5 outperforms blends and diesel in all conceivable scenarios.

The maximum BTE for the LPWO5 blend (34.98%) is the greatest among the other LPWO blends and is 0.725% greater than that of diesel fuel when the maximum load is present for CR 18:1. Calorific value is one factor that affects BTE, and LPWO5 has a calorific value that is similar to clean diesel. The fuel blend’s lower viscosity aids in improved atomization, vaporization, and combustion, which improves BTE. Additionally, the presence of oxygen in the form of esters aids in enhancing fuel combustion, which increases power production^12^. Baranitharan et al.^24^ reported that a higher CR (17.5:1) combined with high-load operation yielded the maximum BTE (22.01%), which was attributed to the increase in in-cylinder pressure and temperature when using 20% AM pyrolysis oil.

**Fig. 8:**
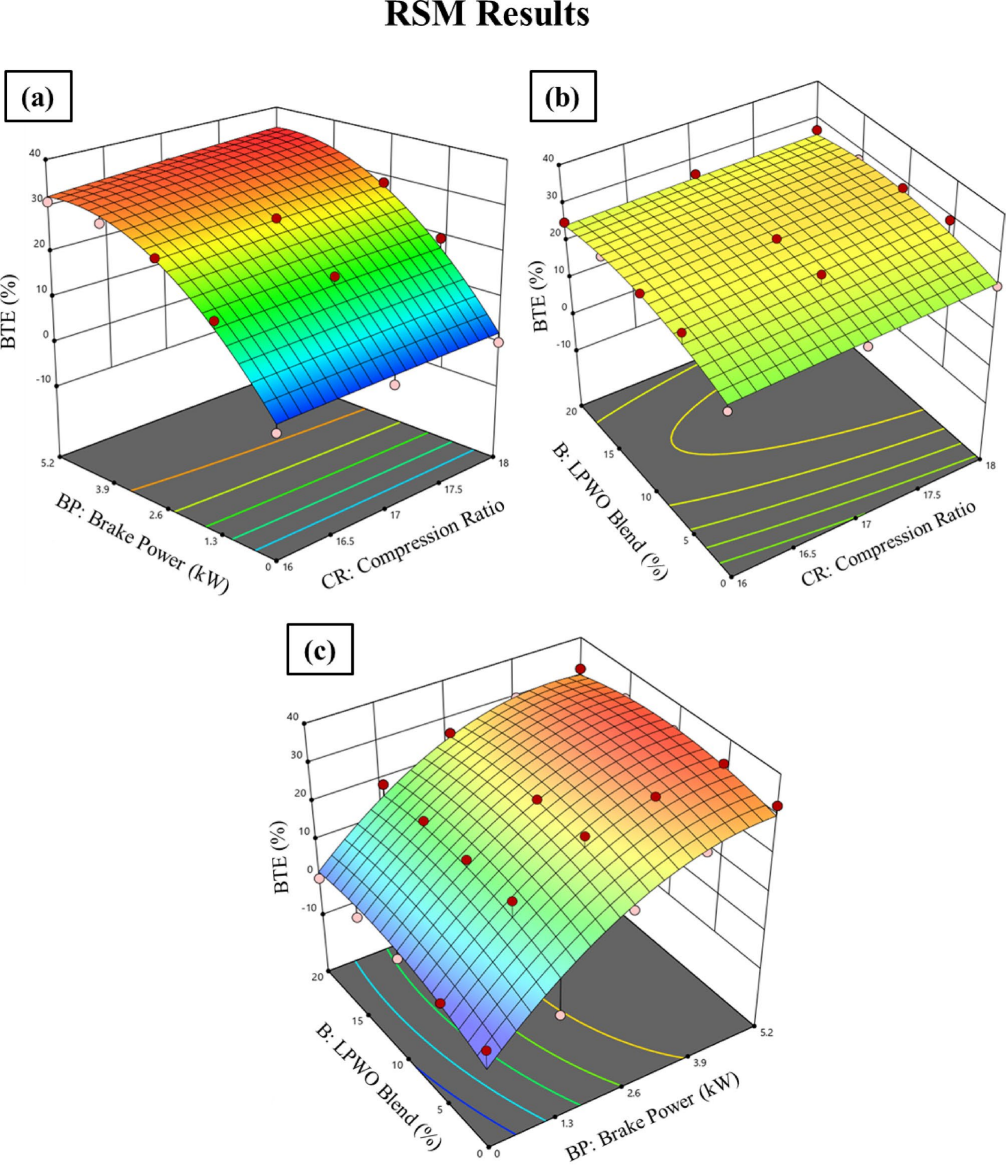
RSM results with 3D surface graphs of **(a)** CR vs. B, **(b)** CR vs. BP, and **(c)** BP vs. CR for BTE.

By maintaining the LPWO blend ratio (B) constant, Fig. 8**(a)** shows the fluctuation in brake power (BP) and compression ratio (CR) for BTE. BTE has been seen to rise with BP and CR due to improved combustion and energy conversion. Higher BP enhances efficiency under load, while higher CR boosts thermal efficiency through increased pressure and temperature, resulting in better fuel utilization^50^. Figure 8**(b)** illustrates the fluctuation in B and CR for BTE while maintaining a constant load (BP). Observations show that BTE rises with CR and falls with the LPWO blend. The rise in BTE with CR is due to higher in-cylinder temperatures and pressures, which enhance combustion and thermal efficiency. However, increasing the LPWO blend reduces BTE because biofuel typically has a lower calorific value than diesel, leading to less efficient combustion and increased fuel consumption^51^. By keeping the CR constant, Fig. 8**(c)** shows how the LPWO blend and load for BTE might vary. According to observations, BTE rises with load and falls with LPWO blend. The iterative statistical regression of BTE with the RSM technique is shown in Eq. (3). It presents the reduced quadratic regression model obtained after excluding statistically insignificant predictors.

3 $$\:\text{B}\text{T}\text{E}\:\left(\text{\%}\right)=5.2899\:\text{C}\text{R}\:+\:9.1289\:\text{B}\text{P}\:+\:1.4603\:\text{B}\:-\:0.0609\:\text{B}\text{P}\text{*}\text{B}\:-\:1.3577\:{\text{B}\text{P}}^{2}-\:0.0370\:{\text{B}}^{2}-53.14$$

#### Brake-specific fuel consumption (BSFC)

**Fig. 9:**
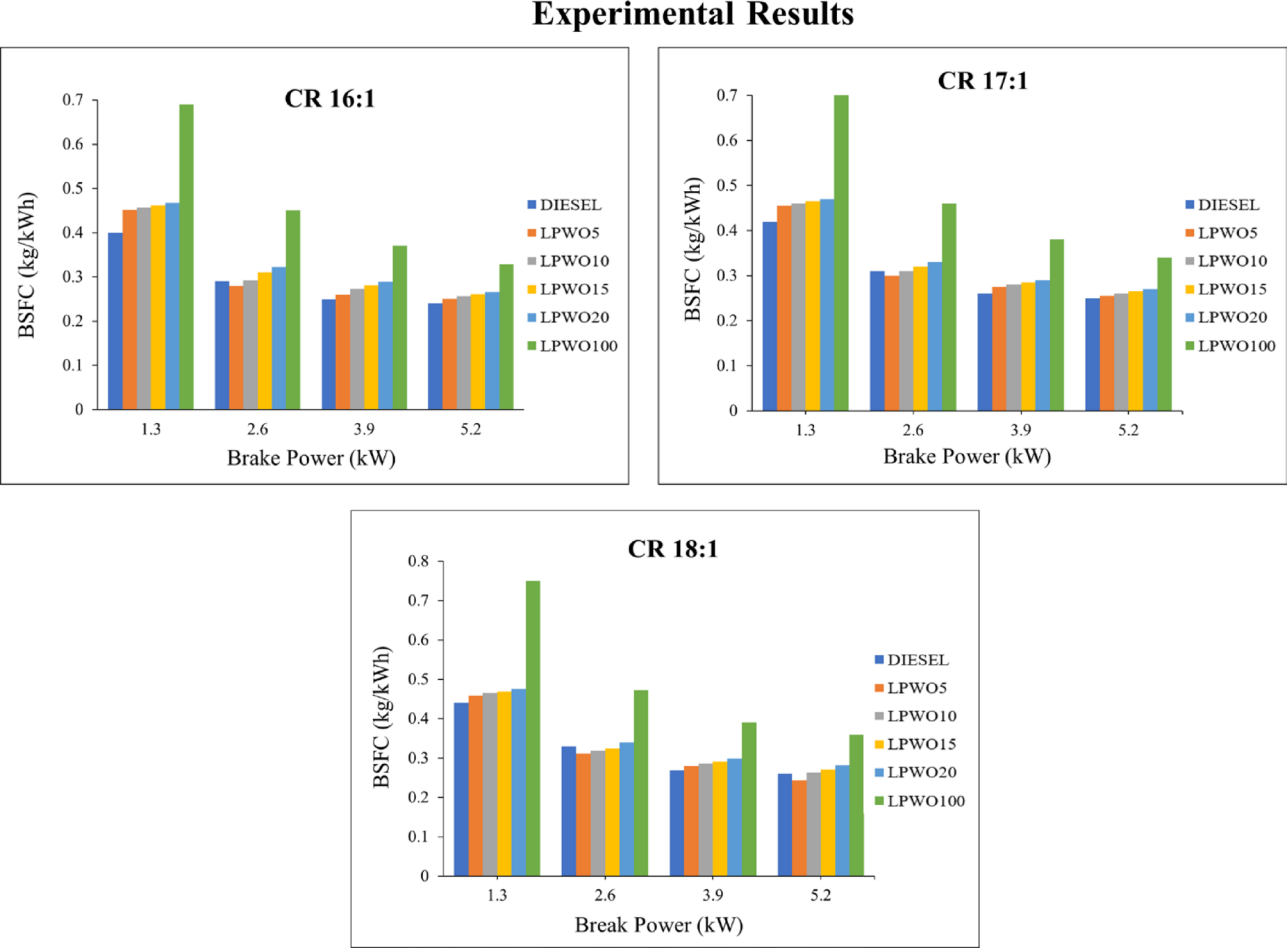
Experimental results of BSFC with respect to BP at CRs of 16:1, 17:1, and 18:1 for diesel and different LPWO blend ratios.

One of the most crucial factors to take into account when evaluating performance is engine economy, and it is examined using BSFC. The fuel used to produce unit power is referred to as BSFC^40^. The engine’s ability to convert fuel into productive work is inversely correlated with the BSFC rating. It indicates that for an individual CR, BSFC decreases with an increase in loads, but LPWO blends consume more fuel than neat diesel fuel. Additionally, the BSFC is increased for a subsequent increase in brake power by changing the CR from 16:1 to 18:1. At maximum load, LPWO5’s minimum BSFC was found to be 0.243 for CR 18:1, 0.251 for CR 16:1, and 0.255 for CR 17:1, which is shown in Fig. 9. Among this lowest fuel consumption is witnessed in LPWO5 for compression ratio of 18:1 at 5.2 kW. This is because LPWO5 mixes and evaporates with air molecules more effectively than diesel does, because it has a low boiling point, and LPWO5’s lower viscosity speeds up the atomization process. The presence of inherent oxygen promotes rapid and thorough combustion, even with a slightly low net calorific value^20^.

**Fig. 10:**
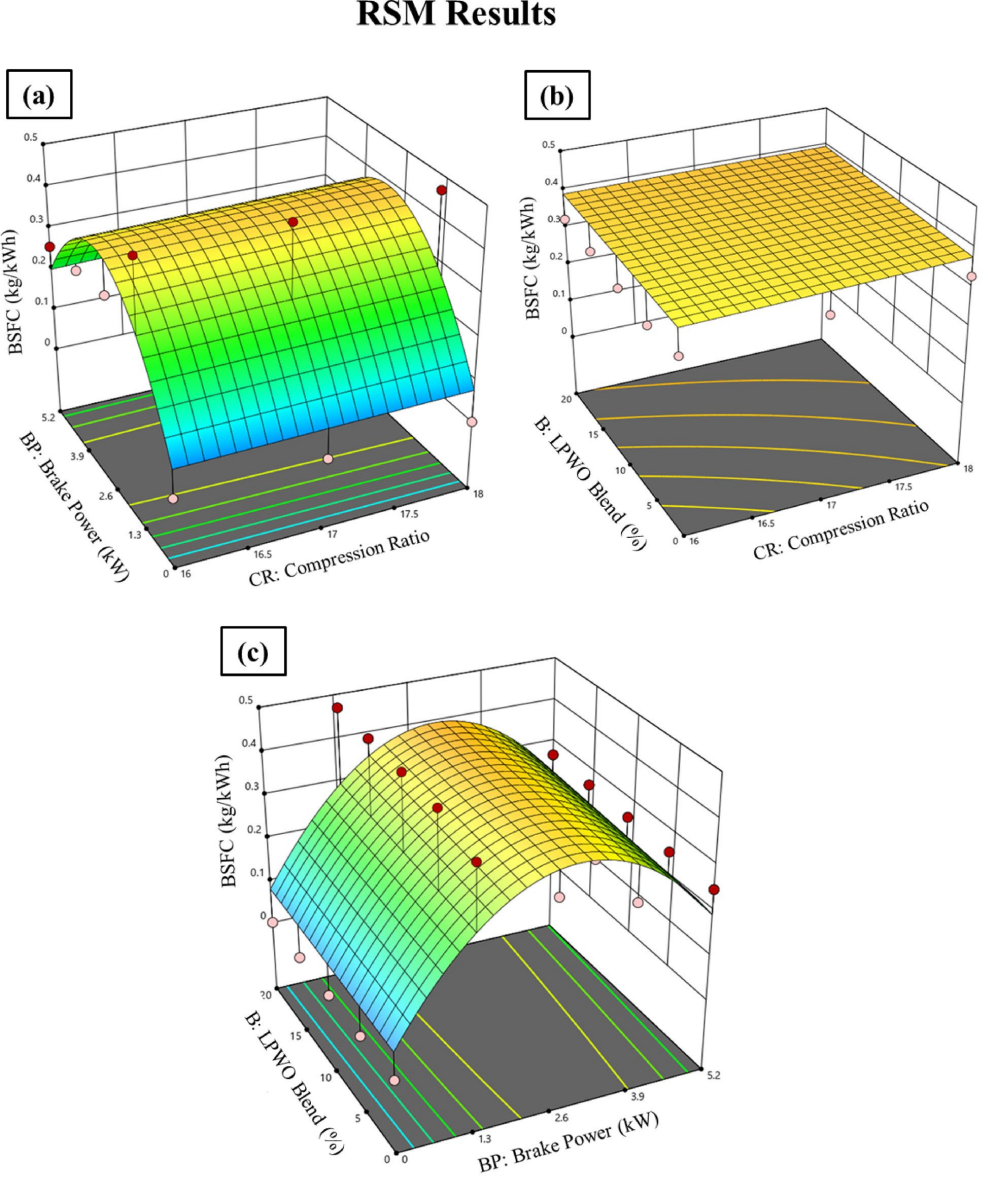
RSM results with 3D surface graphs of **(a)** CR vs. B, **(b)** CR vs. BP, and **(c)** BP vs. CR for BSFC.

The fluctuation in load (BP) and CR for BSFC while maintaining the B constant is seen in Fig. 10**(a).** It has been noted that CR is marginally increased by BSFC and that BSFC grows to 2.6 kW before decreasing when the LPWO blend is used. By maintaining the load constant, Fig. 10**(b)** displays the fluctuation in LPWO blend percentage and CR for BSFC. It has been shown that BSFC decreases with CR and LPWO blend percent. A similar trend was reported by Patel et al.^25^, where the BSFC value showed a decreasing pattern with an increase in CR and the blending proportion of Jatropha curcas shell bio-oil in diesel. By maintaining the CR constant, Fig. 10**(c)** depicts the fluctuation in LPWO blend and load for BSFC. It has been shown that BSFC rises with LPWO blend up to 2.6 kW before falling. This trend occurs because at lower loads, the lower calorific value of the LPWO blend leads to incomplete combustion, resulting in higher fuel consumption. As the load increases beyond 2.6 kW, combustion improves due to higher in-cylinder temperatures and better atomization, which enhances fuel utilization and reduces BSFC^51^. The RSM approach with regression expression for BSFC is the refined quadratic model, excluding non-significant factors, which is formulated in Eq. (4).

4 $$\:\text{B}\text{S}\text{F}\text{C}\:\left(\text{k}\text{g}/\text{k}\text{W}\text{h}\right)=0.1935\:\text{B}\text{P}\:+\:0.0066\:\text{B}-\:0.0003\:\text{C}\text{R}\text{*}\text{B}-\:0.0353\:{\text{B}\text{P}}^{2}-0.0946$$

#### Exhaust gas temperature (EGT)

**Fig. 11:**
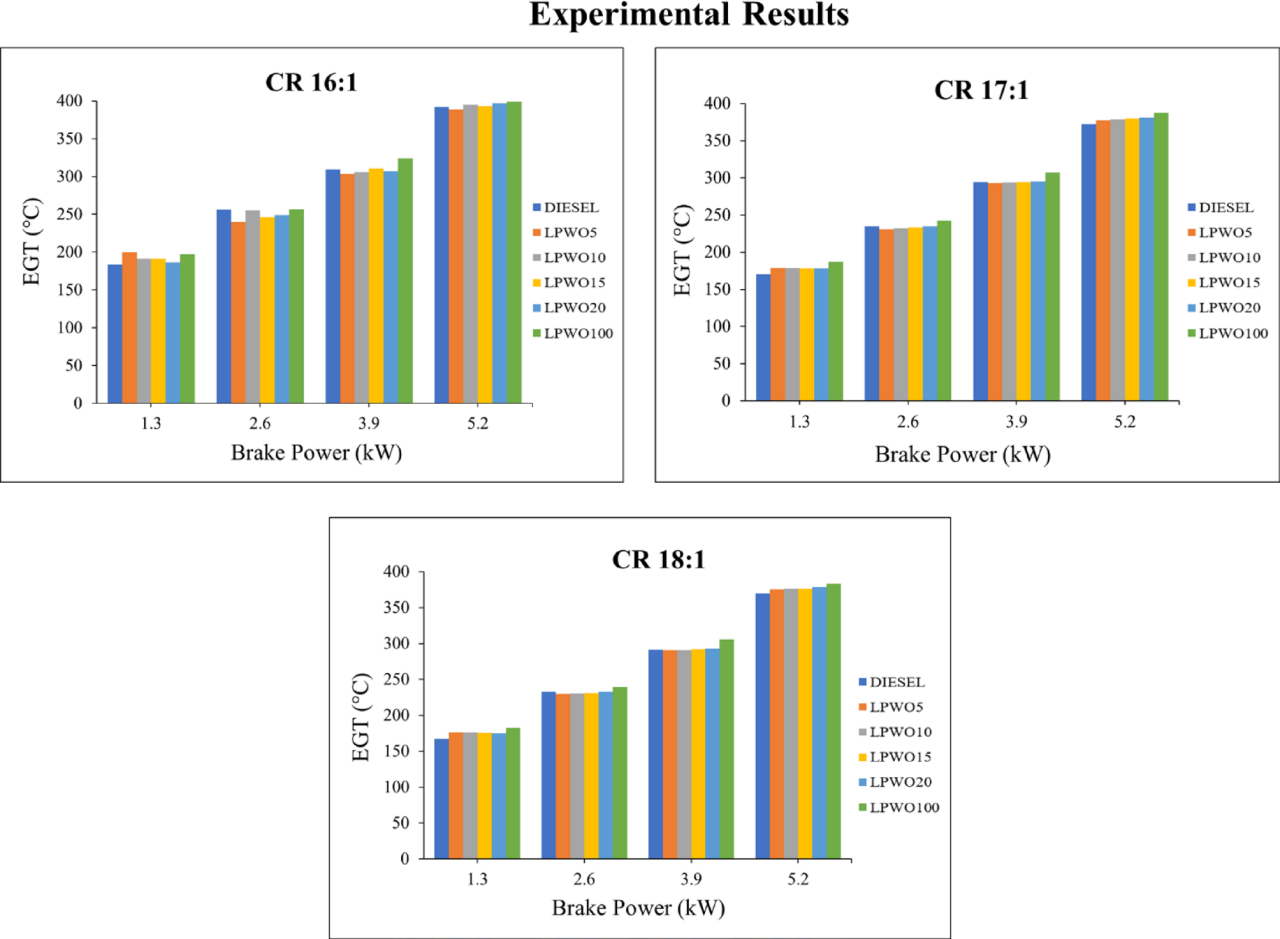
Experimental results of EGT with respect to BP at CRs of 16:1, 17:1, and 18:1 for diesel and different LPWO blend ratios.

EGT is a crucial factor in determining how efficiently a diesel engine performs; the lower the EGT is for a particular speed and load, the more effectively the engine is operating. Figure 11 displays the variation of EGT at different BP operating under compression ratios 16:1, 17:1, and 18:1. It has been demonstrated that for all fuels, rising BP is followed by increasing EGT. When compared to plain diesel, EGT for LPWO blends is higher and rises when the amount of lemon peel oil is increased. When operating at varied CRs, the LPWO100’s EGT values are 390.17 °C, 387.51 °C, and 383.17 °C at maximum load. This could be a result of LPWO100 creating more EGT than pure diesel fuel because of its higher oxygen(O_2_) concentration. This rise in EGT reflects greater fuel use. For plain diesel and LPWO mixes, the diesel engine’s greater 18:1 compression ratio results in lower EGT. This could be a result of air being sucked in during the suction stroke at a greater compression ratio, which raises the temperature of the incoming air. The complete combustion of the fuel and a reduction in EGT are both facilitated by the greater air temperature, which also encourages improved air-fuel mixing and fuel atomization^52^.

**Fig. 12:**
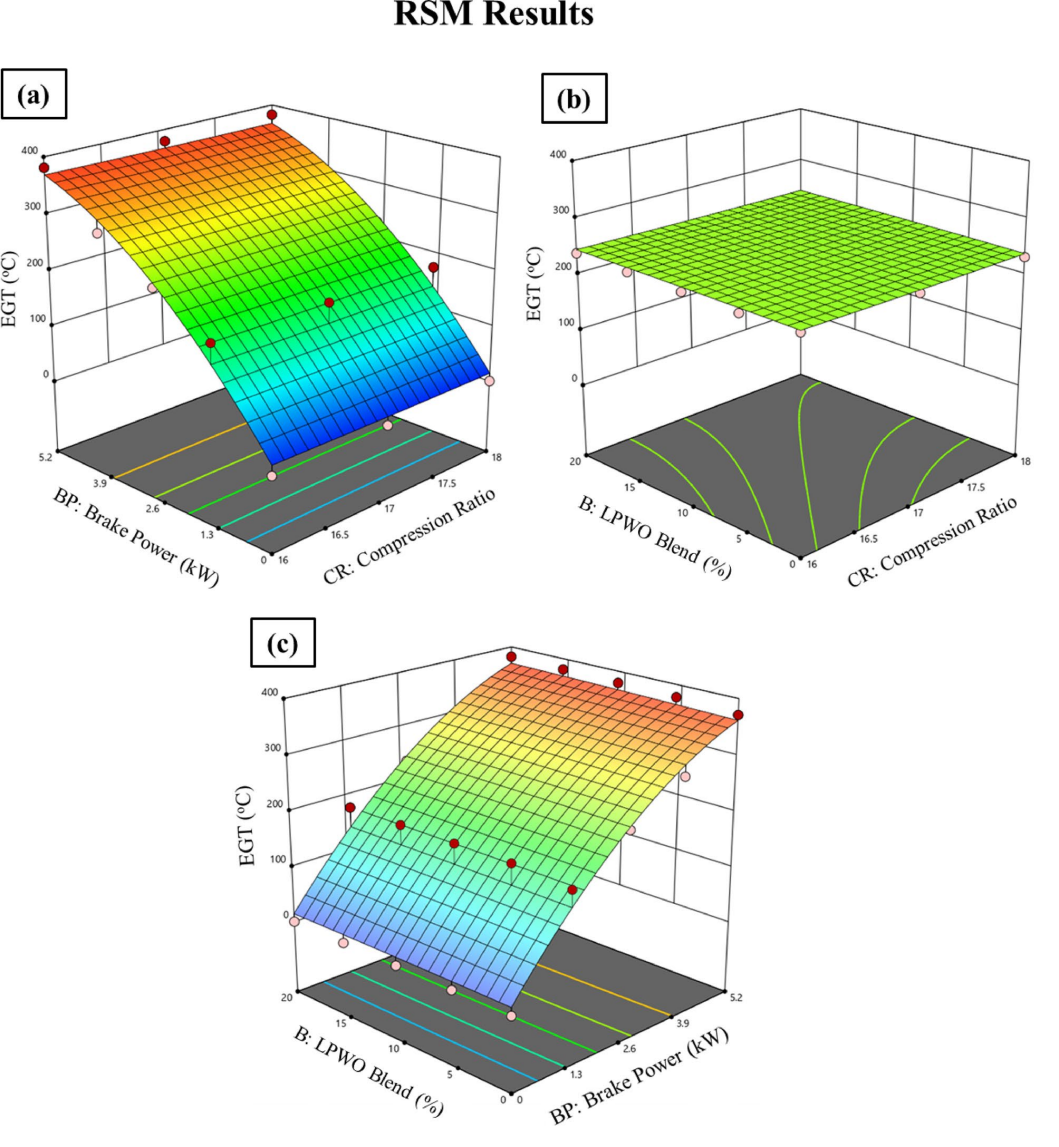
RSM results with 3D surface graphs of **(a)** CR vs. BP, **(b)** CR vs. B, and **(c)** BP vs. B for EGT.

By maintaining the LPWO blend (B) constant, Fig. 12**(a)** indicates the fluctuation in compression ratio (CR) and load (BP) for EGT. It has been found that both CR and load cause a small drop in EGT. Keeping the load constant, Fig. 12**(b)** exhibits the fluctuation in the blend percentage of CR and LPWO for EGT. EGT is seen to decrease as the LPWO blend and CR increase. This is because higher CR leads to more efficient combustion and better energy extraction, leaving less heat in the exhaust. Additionally, higher LPWO blends, due to their lower calorific value and slower combustion, result in reduced peak flame temperatures, further lowering EGT^50^. According to Kannan et al.^30^, the EGT of the 20% tectona grandis biodiesel blend (TB20) was found to be lower than that of blends with higher concentrations. This reduction was attributed to the lower viscosity and intrinsic oxygen content of TB20, which facilitated improved combustion. By keeping the CR constant, Fig. 12**(c)** displays the way the load and LPWO blend for EGT might vary. Evidence suggests that EGT increases with load and decreases with LPWO blend percentage. Based on the significance test results, Eq. (5) expresses the reduced EGT quadratic model with only the significant terms retained.

5 $$\:\text{E}\text{G}\text{T}\:\left({}^{o}C\right)=107.8816\:\text{B}\text{P}+\:0.0596\:\text{B}\text{P}\text{*}\text{B}-\:7.6275\:{\text{B}\text{P}}^{2}+298.46$$

### VCR engine emission characteristics

#### Carbon monoxide (CO) emission

**Fig. 13:**
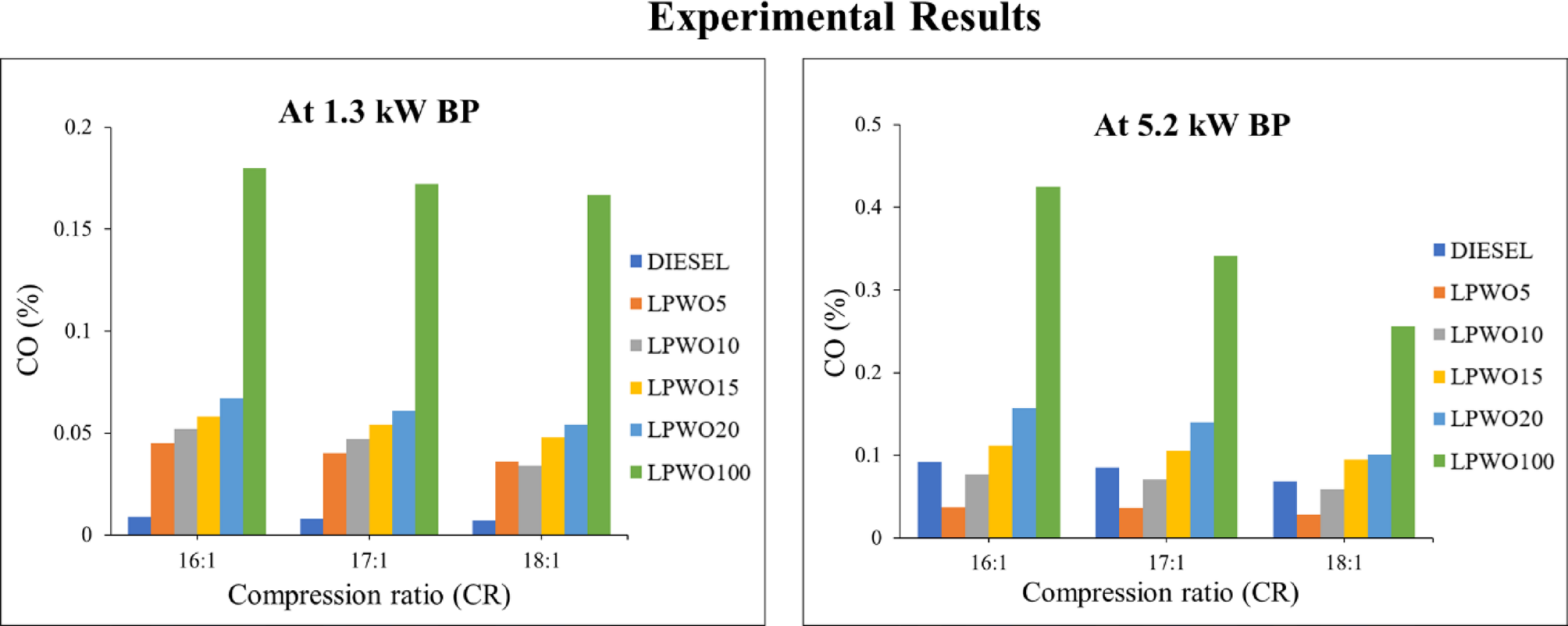
Experimental results of CO emission at 1.3 kW and 5.5 kW BP for varied CRs of diesel and different LPWO blend ratios.

Incomplete or insufficient combustion inside the combustion chamber of a diesel engine commonly results in CO emissions. This can be brought on by a lack of oxygen in the cylinder or an incorrect mixture of air and fuel during an oxidative chemical reaction^21^. The amount of air and fuel that enters the cylinder outside of the chemically optimal ratio also affects how much carbon monoxide (CO) is produced. Figure 13 shows the fluctuations in CO emission for compression ratios 16:1, 17:1, and 18:1 under minimal load and maximum load. For both 1.3 kW and 5.2 kW loads, CO shows a decreasing trend as the CR increases from 16:1 to 18:1. The percentage of emission at low load is higher than that of diesel. But the CO emission of LPWO5 and LPWO10 is less than that of diesel for the maximum load. CO percentages of LPWO5 are 0.0372, 0.0365, and 0.028 for compression ratios 16:1, 17:1, and 18:1. With a high load condition with CR 18:1 operating, it is observed that CO emission decreases by 59.42%. With better atomization of the fuel-air mixture due to optimizing injection delay, high cylinder temperature, and pressure, less CO formation is attained at CR 18:1. Because of the intrinsic oxygen and low viscosity of the fuel, which promotes the atomization and mixing of the fuel with the air and leads to complete combustion and the production of CO_2_ instead of CO ^54,56^.

**Fig. 14:**
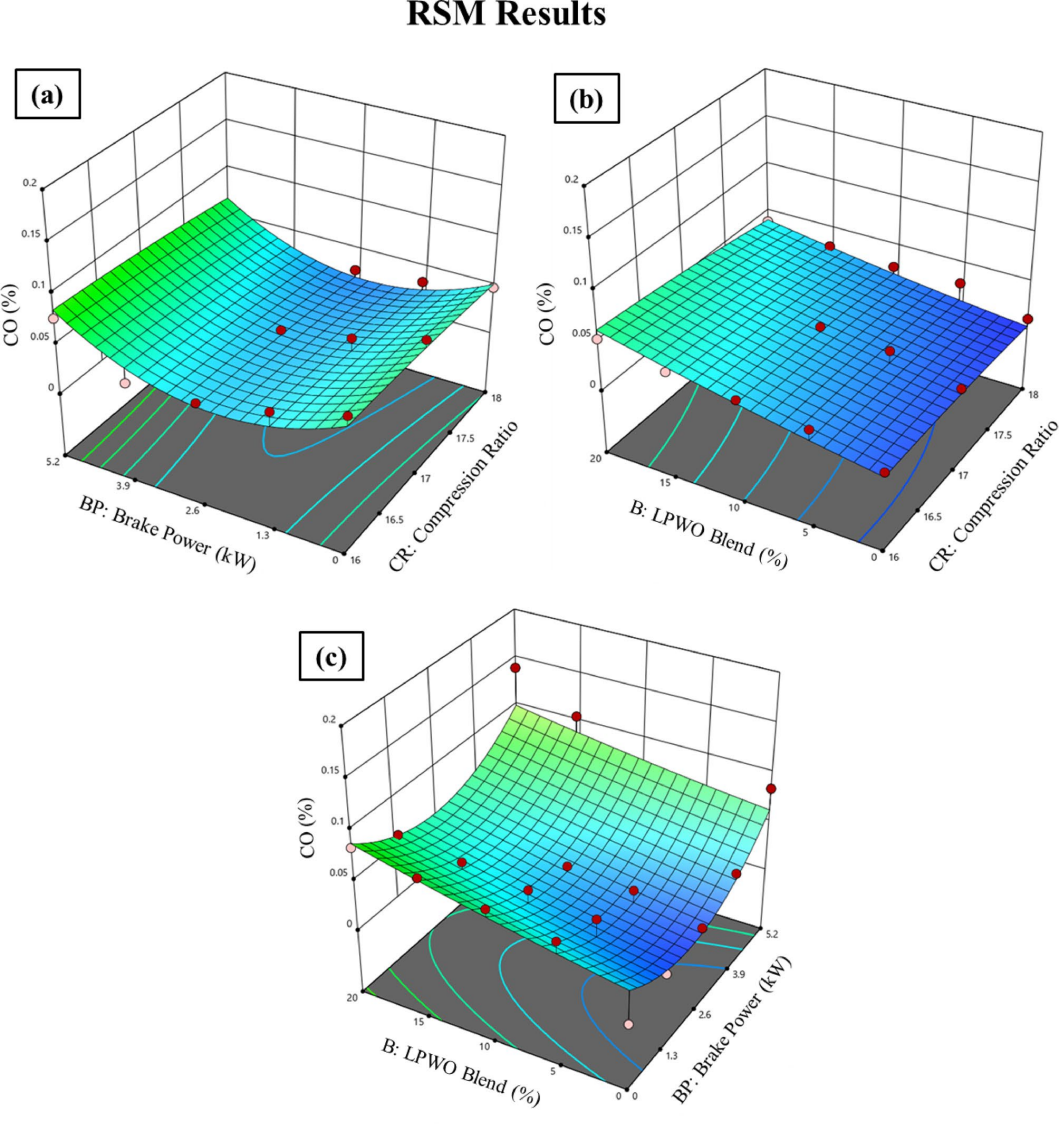
RSM results with 3D surface graphs of **(a)** CR vs. BP, **(b)** CR vs. B, and **(c)** BP vs. B for CO emission.

When the LPWO blend (B) percentage is kept constant, Fig. 14**(a)** demonstrates the fluctuation in compression ratio (CR) and load (BP) for CO. With CR and load, CO is seen to reduce somewhat before increasing after decreasing to 2.6 kW. Keeping the load (BP) constant, Fig. 14**(b)** reveals the way the fraction of CR and LPWO blend changes for CO. It has been shown that CR causes a drop in CO and an increase in the LPWO blend percentage. The reduction in CO with higher CR is due to enhanced combustion efficiency and higher in-cylinder temperatures, which promote more complete oxidation of carbon. In contrast, increasing the LPWO blend raises CO emissions because of the biofuel’s lower volatility, which can lead to incomplete combustion and increased formation of CO^57^. By maintaining the CR constant, Fig. 14**(c)** presents the load and LPWO blend variation for CO. It has been noted that CO rises with the amount of LPWO blend and that CO reduces with load until 2.6 kW, at which point it rises. Equation (6) illustrates the simplified quadratic model OF CO emission derived by removing terms that did not meet the statistical significance threshold.

6 $$\:\text{C}\text{O}\:\left(\%\right)=0.0758\:\text{C}\text{R}-0.0156\:\text{B}\text{P}+0.0086\:\text{B}-0.0003\:\text{C}\text{R}\text{*}\text{B}+0.000023\:{\text{B}}^{2}-0.5969$$

#### Hydrocarbon (HC) emission

**Fig. 15:**
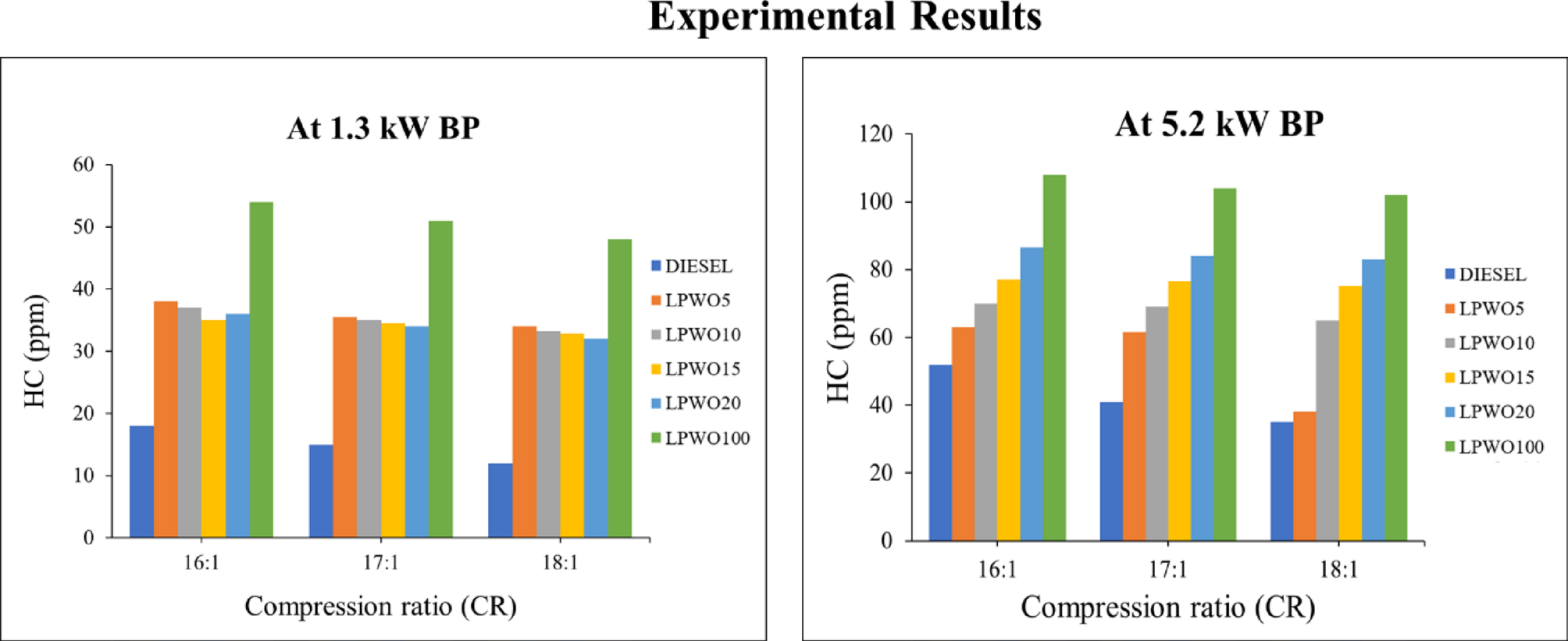
Experimental results of HC emission at 1.3 kW and 5.5 kW BP for varied CRs of diesel and different LPWO blend ratios.

Diesel engine HC emissions can be produced by incomplete combustion, low fuel quality, improper fuel injection time or pressure, and excessive exhaust gas recirculation^54^. As seen in Fig. 15, loading and operating at compression ratios of 16:1, 17:1, and 18:1 result in higher hydrocarbon emissions than diesel fuel emissions for all test fuels. The production of HC increases noticeably as brake power and CR increase because LPWO has a lower cetane number and longer ignition delays. At top load condition operated at 18:1, the value for LPWO5 was found to closely match that of pure diesel of 38.20 ppm, which is 9.14% more than that of diesel. Parida et al. reported that, with a 20% Argemone mexicana biodiesel-diesel blend, the delay period decreases as CR increases, leading to improved combustion and reduced HC emissions.

**Fig. 16:**
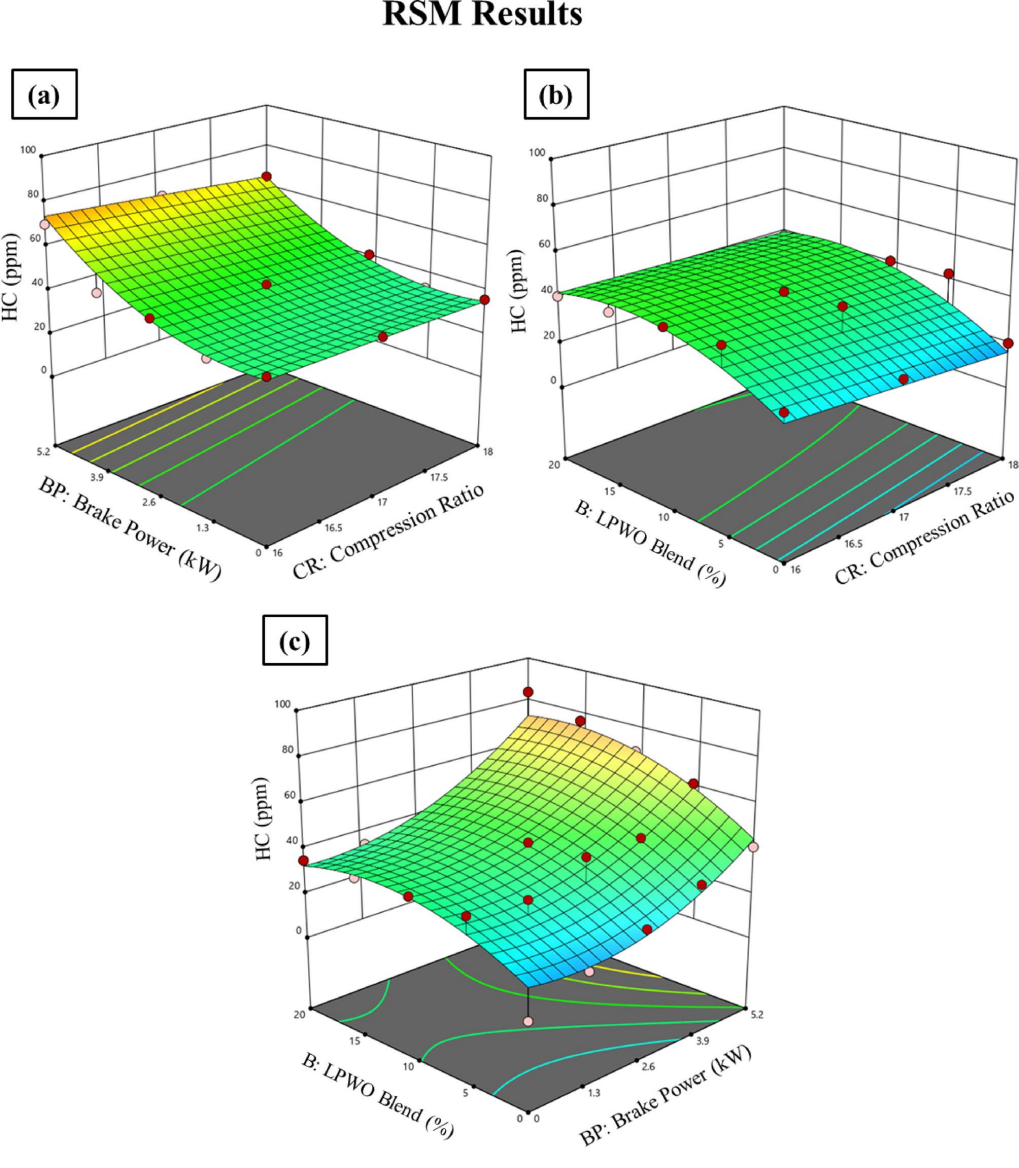
RSM results with 3D surface graphs of **(a)** CR vs. BP, **(b)** CR vs. B, and **(c)** BP vs. B for HC emission.

The graph presented in Fig. 16**(a)** shows how hydrocarbon (HC) emissions vary with changes in compression ratio (CR) and load while maintaining a constant LPWO blend percentage. The statistics show that raising both the CR and the load resulted in increased HC emissions. Figure 16**(b)** illustrates the connection between the compression ratio (CR) and LPWO blend (B) for HC emissions while keeping the load (BP) constant. The rise in HC with increasing CR may be due to fuel quenching near cylinder walls at higher pressures, leading to unburnt hydrocarbons. On the other hand, increasing the LPWO blend reduces HC emissions because the oxygenated nature of the LPWO promotes more complete combustion, thereby lowering unburnt hydrocarbon formation^55^. For HC emissions, Fig. 16**(c)** displays the connection between the load and LPWO blend percentage while maintaining a constant CR. According to the graph, HC emissions rise as load values rise and fall as LPWO blend percentages rise. Following ANOVA screening, the quadratic model of HC emission was reduced to exclude insignificant terms, as shown in Eq. (²² 7).

7 $$\:\text{H}\text{C}\:\left(\%\right)=1.58\:\text{C}\text{R}+6.4713\:\text{B}\text{P}+0.2768\:\text{B}+0.13959\:\text{B}\text{P}{*}\text{B}+1.6796\:\text{B}\text{P}^2-0.1081\:\text{B}^2+28.44$$

#### Nitrogen oxides (NO_x_) emission

**Fig. 17:**
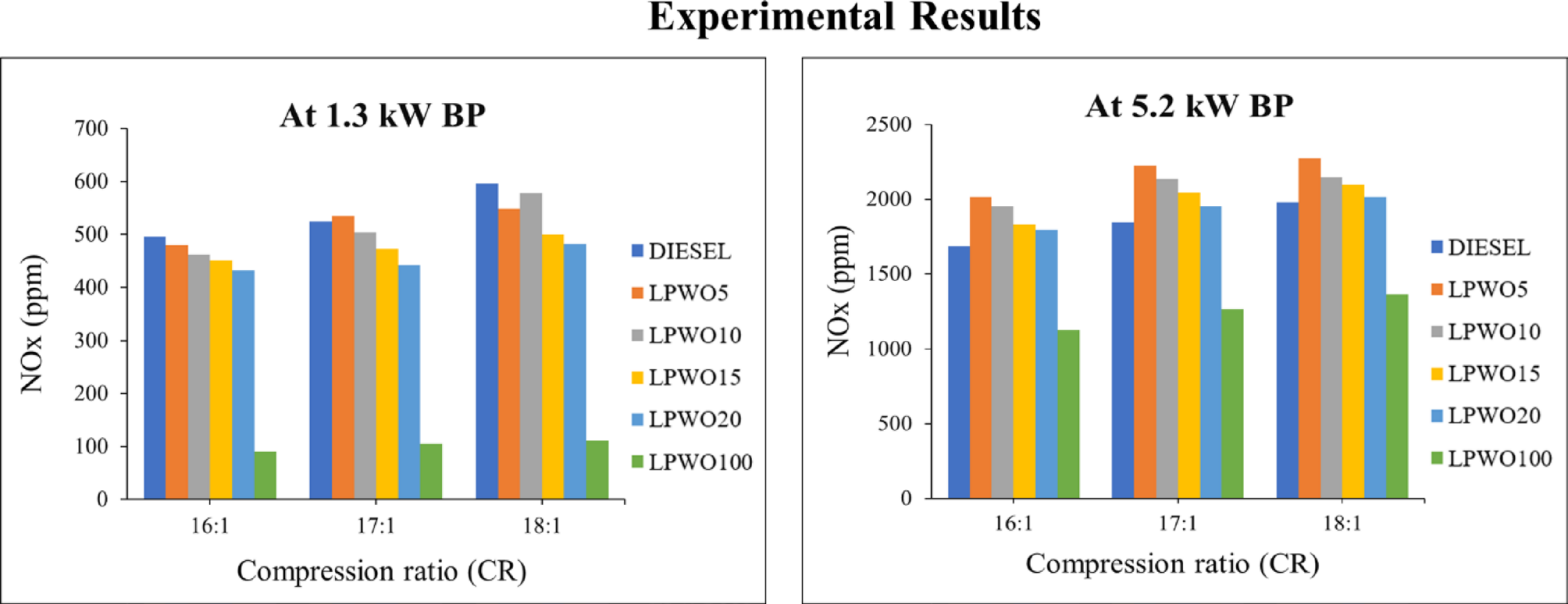
Experimental results of NO_x_ emission at 1.3 kW and 5.5 kW BP for varied CRs of diesel and different LPWO blend ratios.

NOx emissions in diesel engines are mainly generated by high combustion temperatures and pressures, which increase the generation of nitrogen oxides. The combustion of air’s oxygen and nitrogen also contributes to the creation of NO_x_^56^. According to Fig. 17, all test fuels generate more NOx when their compression ratios are increased. For 1.3 kW BP, at every compression ratio, LPWO blends emit less NO_x_ than neat diesel. Whereas at top load conditions, Nitrogen oxide production is more than that of the diesel engine due to higher in-cylinder temperature. While NO_x_ is kept as low as feasible during low-load engine runs by localized in-cylinder gas temperature, the VCR setup running at CR 16:1 generates less NO_x_ since the flame production temperature is lower. As the amount of LPWO in diesel fuel rises, NO_x_ formation shows a decreasing trend, and when pure LPWO is used, emissions are far less than all the blends and neat diesel. At high BP, the value of LPWO100 for CR 16:1, 17:1, and 18:1 is 33.25%, 31.42%, and 30.99% less than their corresponding diesel values. Due to the LPWO blend’s lower viscosity and cetane number, the ignition delay is longer. The outcome is a rapid rise in the temperature and pressure inside the cylinder. Fuel builds up as a result of delayed ignition. The presence of inherent nitrogen along with these various factors could result in increased NO_x_ emission^20,53^.

**Fig. 18:**
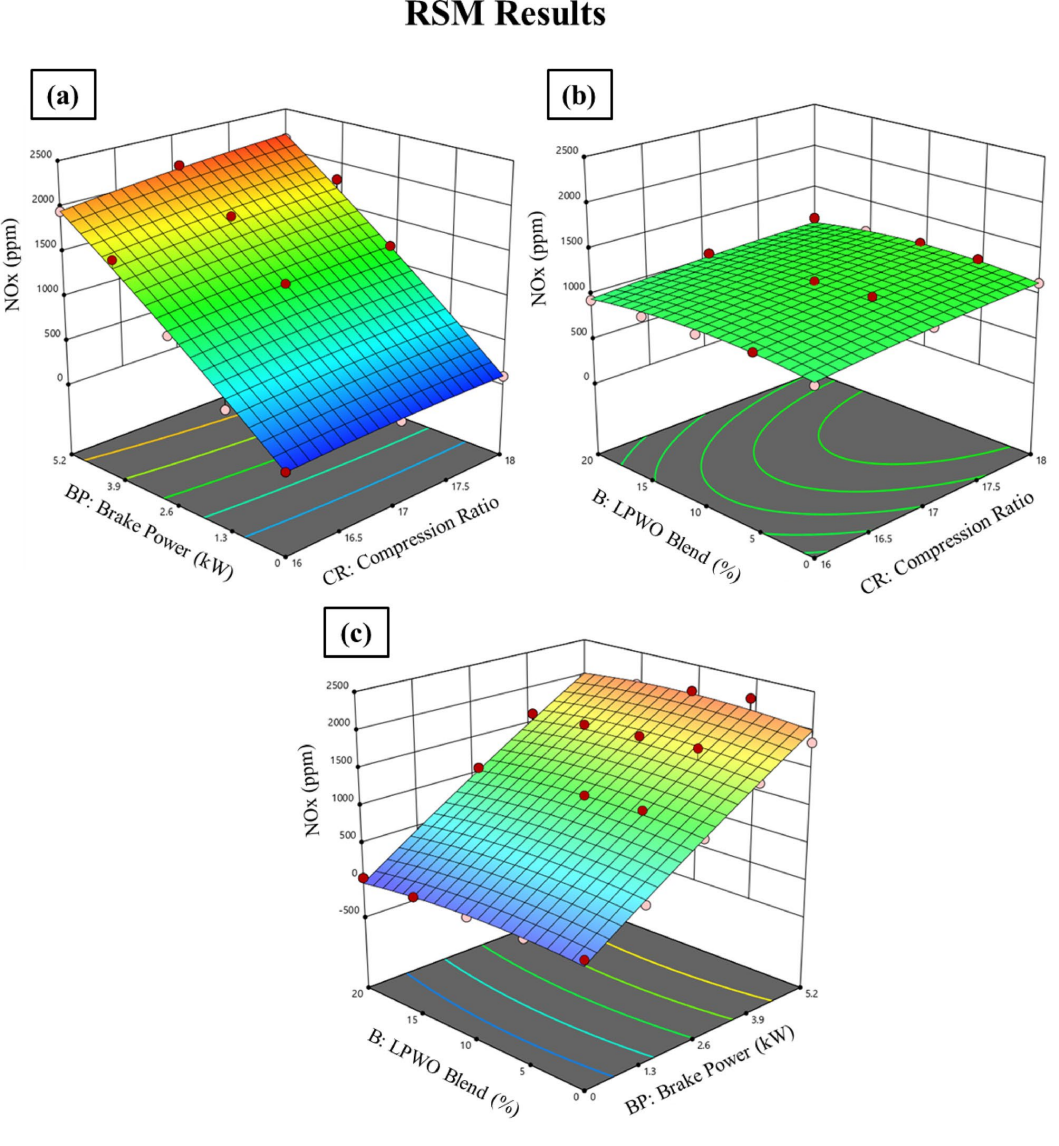
RSM results with 3D surface graphs of **(a)** CR vs. BP, **(b)** CR vs. B, and **(c)** BP vs. B for NO_x_ emission.

When the LPWO blend (B) proportion is kept consistent, Fig. 18**(a)** shows the fluctuation in compression ratio (CR) and load (BP) for NO_x_. It should be remembered that NO_x_ increases with CR and load. By maintaining the load constant, Fig. 18**(b)** shows the fluctuation in the CR and LPWO blend percent for NO_x_. NO_x_ is seen to grow with CR and with LPWO blend percentage, up to LPWO10, before it starts to drop. The rise in NO_x_ with CR is due to higher in-cylinder temperatures and pressures, which favour thermal NO_x_ formation. Similarly, up to LPWO10, the oxygen-rich nature of the blend enhances combustion, raising temperature and promoting NO_x_ formation. However, beyond LPWO10, the higher viscosity and lower calorific value of the blend lead to slower combustion and reduced peak temperatures, resulting in a decline in NO_x_ emissions^29^. By maintaining the CR constant, Fig. 18**(c)** shows the fluctuation in load and LPWO blend for NO_x_. According to research, NO_x_ rises with load and climbs with LPWO mix up to LPWO10 before starting to fall. Equation (² 8) represents the parsimonious quadratic model of NO_x_ emission, constructed by retaining only the influential variables and excluding those deemed insignificant.

8 $$\:{\text{N}\text{O}}_{\text{x}}\:\left(\text{p}\text{p}\text{m}\right)=822.88\:\text{C}\text{R}\:+\:59.74\:\text{B}\text{P}\:+\:20.51\:\text{C}\text{R}{*}\text{B}\text{P}\:+\:1.156\:\text{B}\text{P}{*}\text{B}-\:7.058\:\text{B}\text{P}^2-0.8855\:{\text{B}}^{2}-7116$$

#### Carbon dioxide (CO_2_) emission

**Fig. 19:**
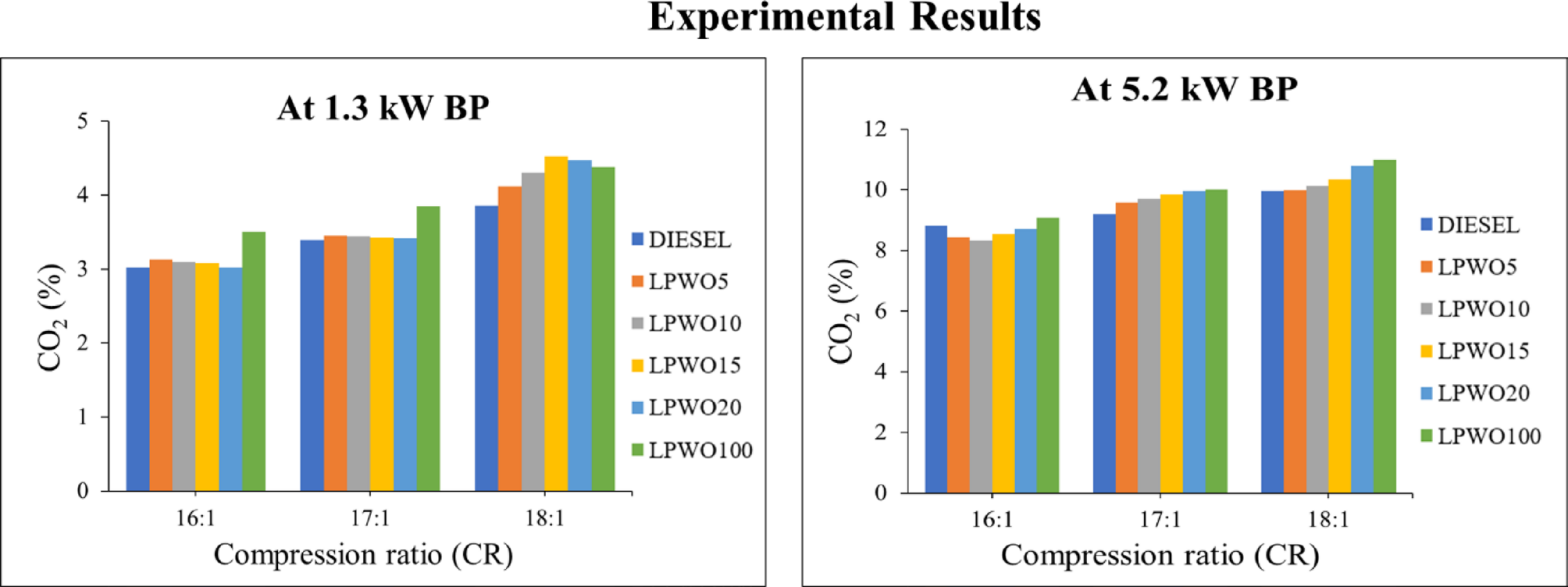
Experimental results of CO_2_ emissions at 1.3 kW and 5.5 kW BP for varied CRs of diesel and different LPWO blend ratios.

Fuel is sprayed into the combustion chamber of a diesel engine, where it reacts with air. The fuel is ignited by the heat of compression, and when it combines with the oxygen in the air, carbon dioxide (CO_2_) and water vapour are produced^22^. Figure 19 demonstrates that raising the CR from 16:1 to 18:1 increases CO_2_ emissions from all fuels significantly. Hence, increasing the LPWO content of diesel fuel enables raising CO_2_ emissions. It’s probable that increasing the quantity of O_2_ in the LPWO blend, which oxidizes CO into CO_2_, may result in a larger output of CO_2_ from pure LPWO combustion. At CR 18:1 with 5.2 kW BP, CO_2_ emissions are increased by 0.201%, 1.504%, 3.814% and 8.325% for LPWO5, LPWO10, LPWO15 and LPWO20, in comparison to diesel. The fuel mixture burning entirely, which is made possible by the existence of intrinsic oxygen in the form of an ester, is the cause of the rise in CO_2_ emissions. Due to LPWO’s low boiling point and low viscosity, the fuel is fully mixed and atomized with the air in the cylinder and also includes unsaturated hydrocarbons, which, when burnt, form CO_2_^55^.

**Fig. 20:**
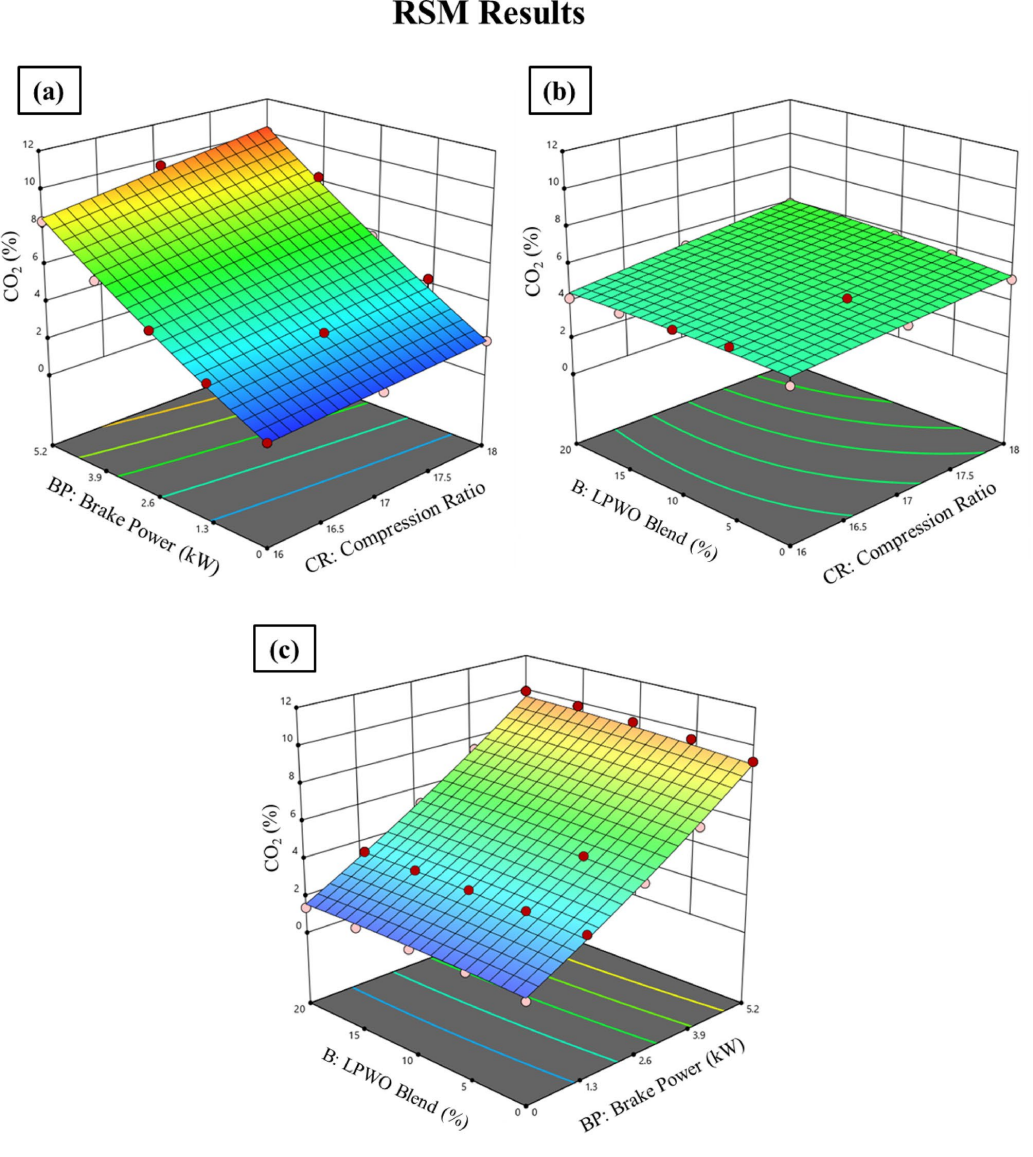
RSM results with 3D surface graphs of **(a)** CR vs. BP, **(b)** CR vs. B, and **(c)** BP vs. B for CO_2_ emission.

Through keeping a constant LPWO blend (B) percentage, Fig. 20**(a)** shows the fluctuation in compression ratio (CR) and load (BP) for CO_2_. It has been found that CO_2_ rises with both CR and load. By maintaining the load constant, the fluctuation in the CR and LPWO blend for CO_2_ is shown in Fig. 20**(b)**. According to observations, CO_2_ rises with CR and falls with LPWO blend percentage. The increase in CO₂ with CR is due to improved combustion efficiency at higher compression, leading to more complete oxidation of carbon in the fuel. In contrast, CO₂ decreases with rising LPWO blend percentage because the lower carbon content and calorific value of the LPWO result in reduced overall carbon combustion, thus lowering CO₂ emissions^50^. Figure 20**(c)** shows the fluctuation in CO_2_ with load and LPWO blend while maintaining a constant CR. According to observations, CO_2_ rises with load and falls with LPWO blend. According to Elkelawy et al.^57^, CO_2_ emissions rise as engine load increases because higher loads encourage more thorough combustion and higher temperatures inside the cylinder. The oxidation of CO to CO_2_ is improved by the increase in combustion temperature, which lowers CO emissions while increasing CO_2_ formation. The optimized quadratic model for CO_2_ emission, derived through stepwise elimination of non-significant terms, is provided in Eq. (9).

9 $$\:{\text{C}\text{O}}_{2}\:\left(\text{\%}\right)=0.3603\:\text{C}\text{R}-0.9637\:\text{B}\text{P}-0.3010\:\text{B}+0.1296\:\text{C}\text{R}\text{*}\text{B}\text{P}+0.0192\:\text{C}\text{R}\text{*}\text{B}\:+\:0.0046\:\text{B}\text{P}\text{*}\text{B}+0.0425\:{\text{B}\text{P}}^{2}-2.41$$

#### Smoke emission

**Fig. 21:**
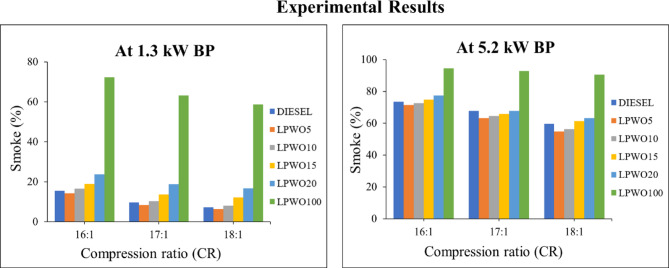
Experimental results of smoke emissions at 1.3 kW and 5.5 kW BP for varied CRs of diesel and different LPWO blend ratios.

Smoke from a diesel engine, which is made up of minute soot, sulfur oxides, and pollutants emitted into the air, is an obvious sign of incomplete combustion^57^. Smoke creation in a CI engine can be produced by a number of circumstances, including low engine temperature, poor fuel quality, wrong fuel injection timing, or an inadequate oxygen supply in the combustion chamber^18^. Smoke is created when unburned fuel particles interact with air and other pollutants. Figure 21 indicates that pure LPWO emits more smoke than the other blends and pure diesel. As the CR increases, smoke emission shows a decreasing trend. However, as the concentration of LPWO increases in diesel fuel, smoke emission increases. LPWO5 shows a significant decrease in smoke than other blends and diesel. LPWO10, LPWO15, and LPWO20 show fluctuating results when compared to diesel. At peak load state, lower smoke levels were obtained with CR 18:1, and it was reduced by 7.89% for LPWO5 and 5.37% for LPWO10 when correlated to diesel fuel. Due to a lower clearance space, the combustion chamber’s temperature and pressure will increase^18^.

**Fig. 22:**
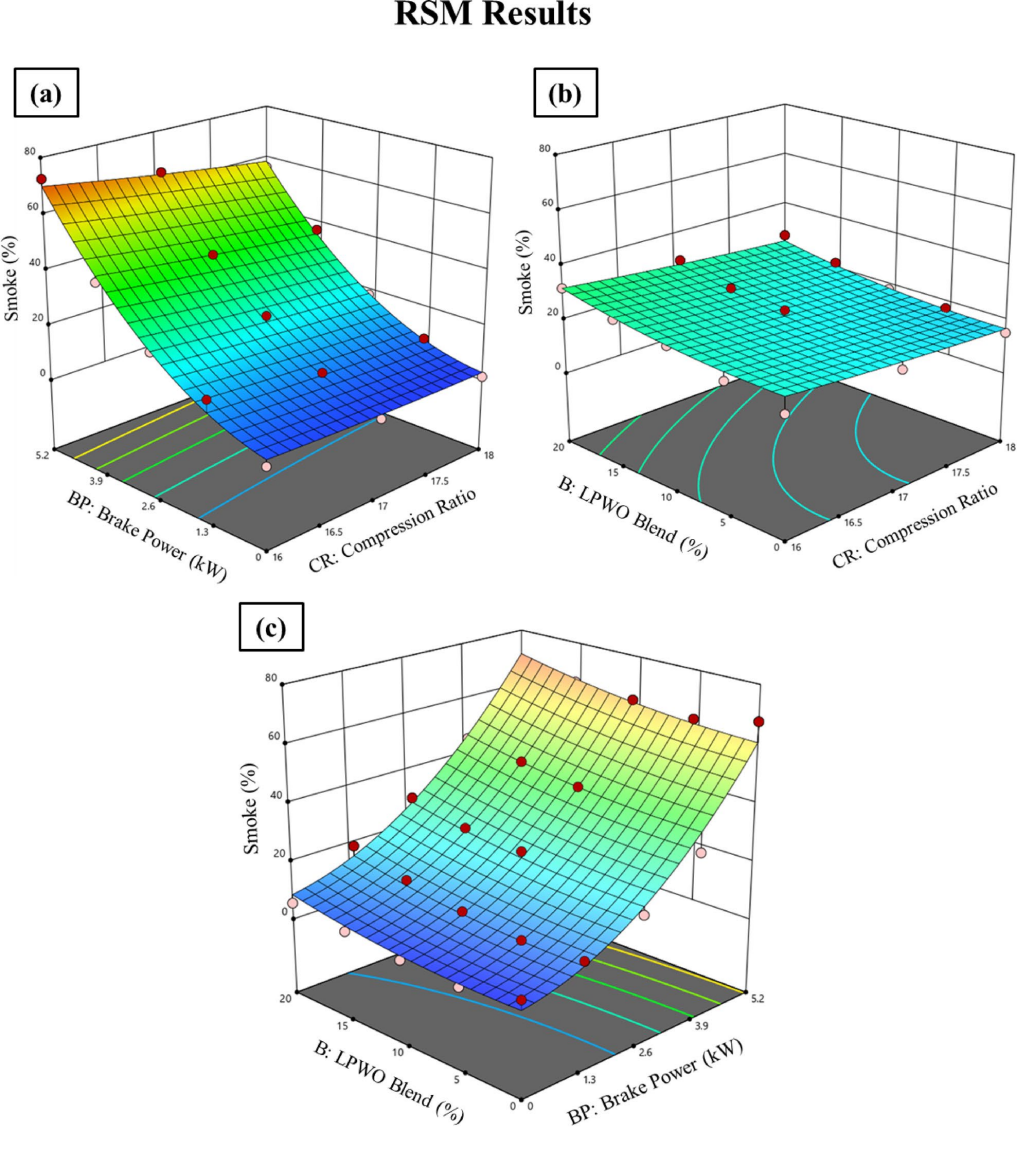
RSM results with 3D surface graphs of **(a)** CR vs. BP, **(b)** CR vs. B, and **(c)** BP vs. B for smoke emission.

Figure 22**(a)** illustrates the changes in CR and BP for smoke by keeping the LPWO blend (B) percentage constant. It is observed that smoke decreases with CR and increases with load. Figure 22**(b)** depicts the variation in CR and LPWO blend percentage for smoke by keeping the load constant. It has been witnessed that smoke decreases with CR and increases with LPWO blend percentage. The reduction in smoke with higher CR is due to better atomization and more efficient combustion resulting from increased thermal and pressure conditions inside the combustion chamber, which reduces soot formation^58^. Conversely, smoke increases with higher LPWO blend percentages because the lower volatility of the LPWO hinders proper fuel-air mixing, leading to incomplete combustion and increased particulate (smoke) emissions^29^. Figure 22**(c)** shows the variation in load (BP) and LPWO blend percentage for smoke by keeping the CR constant. It is noticed that smoke rises with load and LPWO blend percentage. Using CCD-based regression analysis, the insignificant terms were eliminated, and the reduced smoke emission quadratic model is expressed in Eq. (10).

10 $$\:\text{S}\text{m}\text{o}\text{k}\text{e}\:\left(\text{\%}\right)=-17.47\:\text{C}\text{R}+17.29\:\text{B}\text{P}-0.034\:\text{B}-0.90\:\text{C}\text{R}\text{*}\text{B}\text{P}-0.0068\:\text{C}\text{R}\text{*}\text{B}\:+\:0.023\:{\text{B}}^{2}+159$$

### VCR engine combustion characteristics

#### In-cylinder pressure variation

**Fig. 23:**
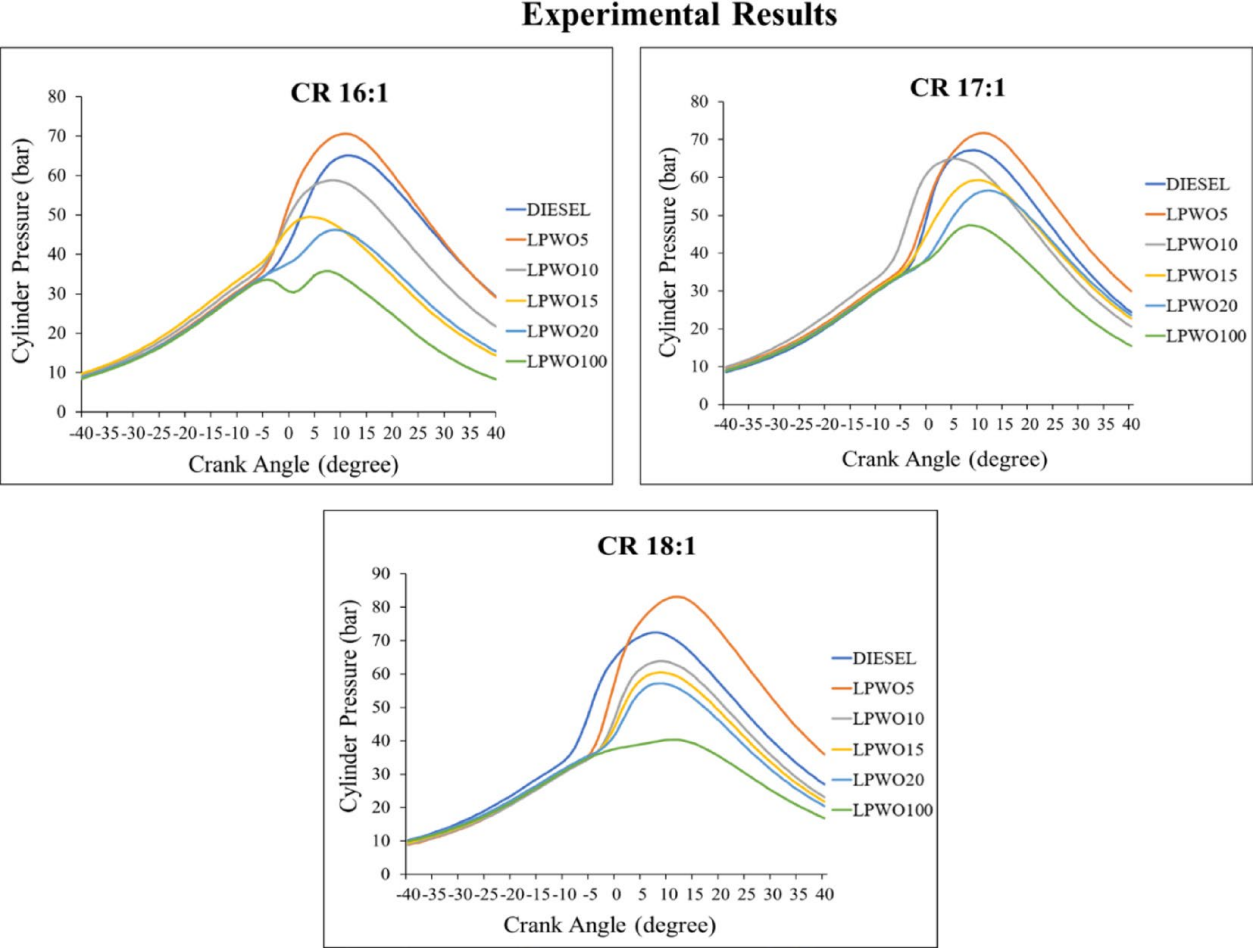
In-cylinder gas pressure vs. crank angle variations for variable CRs of diesel and different LPWO blend ratios.

The work transmission from the gas to the piston may be calculated using data on cylinder pressure. The engine works harder when the in-cylinder pressure is higher^59^. Figure 23 depicts the variation in in-cylinder pressure for compression ratios 16:1, 17:1, and 18:1. As the crank angle increases, in-cylinder pressure for LPWO5 is more than that of neat diesel for CR 16:1. For CR 17:1, LPWO5 shows a better result than neat diesel, whereas LPWO10 comes close to diesel. In-cylinder pressure of LPWO5 in CR 18:1 is greater than the diesel in-cylinder pressure. Peaks in the cylinder curve may be caused by a longer ignition delay^60^. As a result, due to the delay period, combustion proceeds slowly, and fuel accumulates, prompting a rapid rise in pressure at the combustion’s peak. The peak cylinder pressure is found as 83.167 bar for LPWO5 operating at CR 18:1. Muniyappan and Krishnaiah^29^ reported peak cylinder pressures of 72.24 bar for diesel and 71.94 bar for a 20% Mahua biodiesel (M20) blend. The slightly lower cylinder pressure observed with M20 is attributed to its higher density and viscosity, which hinder fuel atomization and evaporation, resulting in less efficient combustion.

#### Heat release rate analysis

**Fig. 24:**
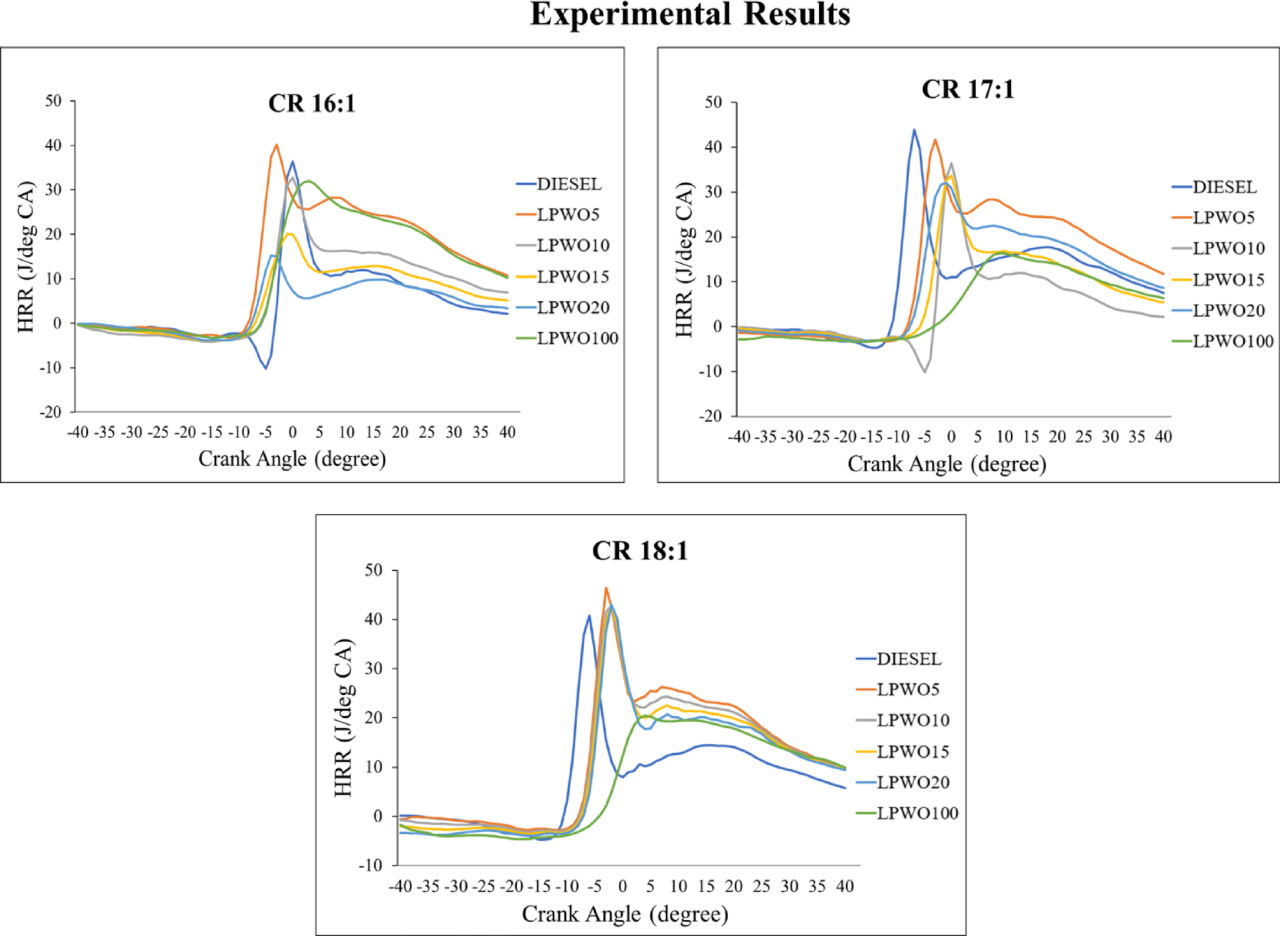
Heat release rate vs. crank angle variations for variable CRs of diesel and different LPWO blend ratios.

The amount of fuel consumed within the engine at any given time impacts its performance, efficiency, and emissions. This rate of fuel combustion is known as the release rate (HRR), and better output is achieved with a higher HRR^50^. Figure 24 depicts the variation in HRR for compression ratios of 16:1, 17:1, and 18:1. With the exception of LPWO5, which has a slightly greater HRR than diesel and peaks before diesel due to the increase in crank angle, other blends have lower HRR than diesel. Whereas in CR 17:1, LPWO5 attains the peak after diesel, the HRR comes close to diesel, still slightly less than diesel. The LPWO5 blend, which is more effective than diesel, has a maximum HRR at a CR 18:1 of 52.6 J/deg. CA. Because of premixed combustion, which is the cause of the higher HRR for lemon peel oil’s lower cetane number, lemon peel oil has a higher HRR^53^. Ashok et al.^68^ observed a similar trend with a low-cetane fuel (lemon peel oil). The addition of lemon peel oil to diesel significantly influenced the HRR by extending the ignition delay period across all load conditions, with the effect being most pronounced at full load.

### DFA-based optimization and experimental verification

**Fig. 25:**
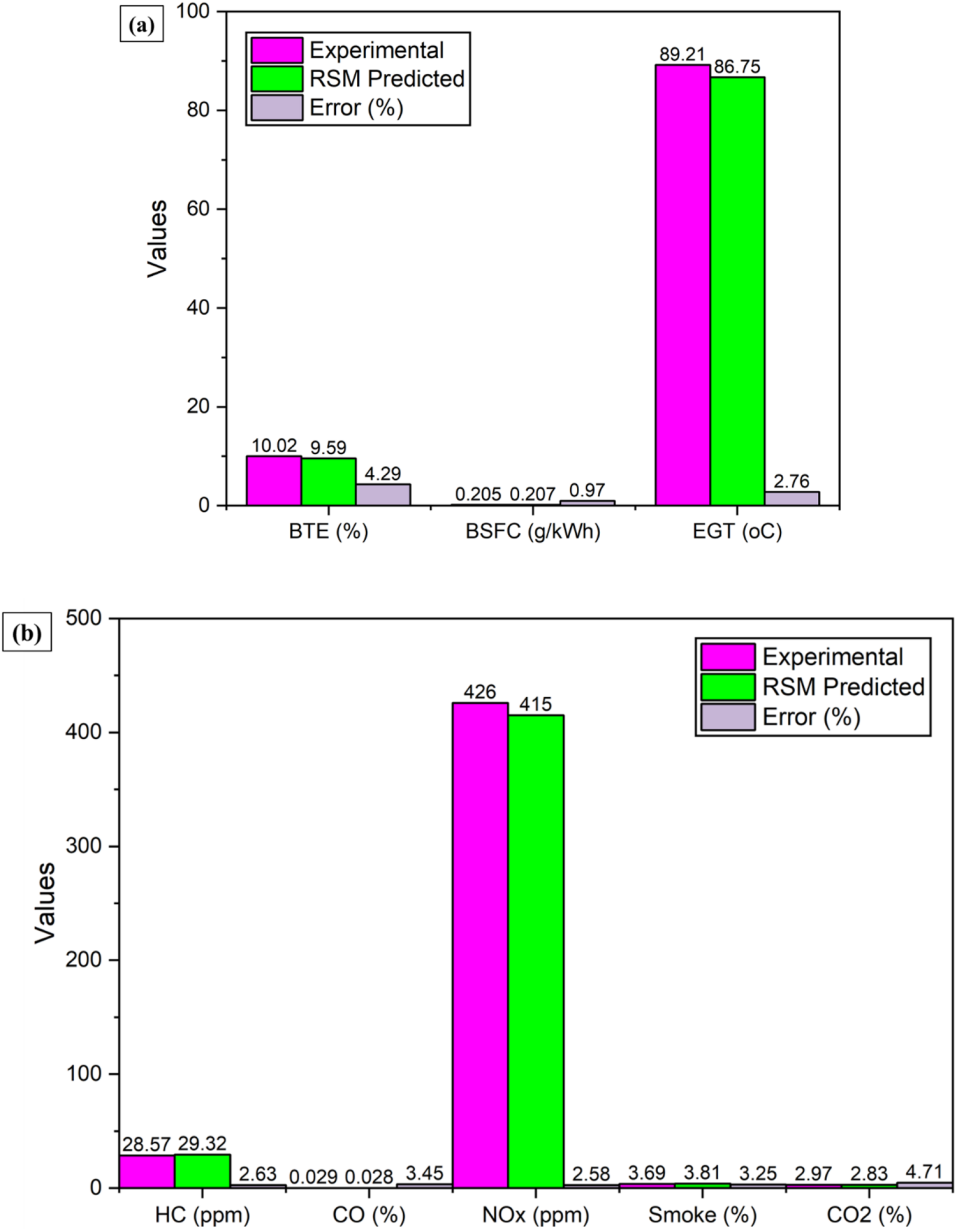
(**a**) Verification of VCR engine performance variables with error. (**b**) Verification of VCR engine emission variables with error.

Table 11 outlines the optimization configuration, which includes the objectives, minimum and maximum thresholds, assigned weights, level of importance, and desirability score for both input parameters and output metrics. The three input variables, compression ratio (CR), brake power (BP), and LPWO blend ratio (B), were set within their specified ranges. Among the output responses, only BTE was targeted for maximization, while all other responses, BSFC, EGT, HC, CO, NOx, smoke opacity, and CO₂, were aimed to be minimized. The individual *(d*_*i*_*)* and overall desirability *(d*_*o*_*)* values being close to 1 indicate the effectiveness of the DFA method. Based on the DFA results, the optimal input conditions determined for this study are a compression ratio of 18:1, a brake power of 1.42 kW, and a 6.51% LPWO blend ratio. The corresponding output response values under these optimized conditions are illustrated in Fig. 25**(a)** and **(b).**

**Table 11:** Constraints for DFA.

Name	Goal	Minimum Limit	Maximum Limit	Lower Weight	Upper Weight	Importance	Desirability
Input variables							
A: Compression ratio (CR)	Range	16:1	18:1	1	1	3	1
B: Brake power (kW)	Range	0 kW	5.2 kW	1	1	3	1
C: LPWO blend ratio (%)	Range	0	20%	1	1	3	1
Output variables (VCR performance)							
BTE (%)	maximize	0	34.985	1	1	3	0.9741
BSFC (kg/kWh)	minimize	0	0.476	1	1	3	0.9523
EGT (^o^C)	minimize	0	383.97	1	1	3	0.9774
Output variables (VCR emission)							
HC (ppm)	minimize	0.001	0.157	1	1	3	0.8299
CO (%)	minimize	3	86.5	1	1	3	0.8476
NO_x_ (ppm)	minimize	35	2276	1	1	3	0.8303
Smoke (%)	minimize	0.8	77.5	1	1	3	0.9606
CO_2_ (%)	minimize	1.22	10.8	1	1	3	0.8321
Combined							0.8997

The accuracy of the optimized responses is confirmed through experimental testing, and the percentage error is determined using Eq. (11)^37^, as shown below.

11 $$\:\left(\%\right)\:error=\left(\frac{VCR\:experimental\:value\:-\:VCR\:predicted\:value\:}{VCR\:experimental\:value}\right)\times\:100$$

Figure 25**(a)** and **(b)** illustrate the experimental validation of the RSM model under VCR engine conditions set at a compression ratio of 18:1, brake power of 1.42 kW, and a 6.51% LPWO blend ratio. Among the output responses, the lowest error was observed for BSFC (kg/kWh) at 0.97%, while the highest was recorded for CO₂ (%) at 4.71%. This confirms that all output errors remain below 5%, demonstrating the reliability of the RSM model with DFA optimization. These results clearly indicate the robustness and accuracy of the RSM in predicting VCR engine performance and emission variables.

Figure 25**(a)** Verification of VCR engine performance variables with error.

## Conclusions

This study explored the use of lemon peel waste for producing lemon peel waste oil (LPWO) and evaluated the performance, combustion, and emission behaviour of LPWO and its blends in a VCR engine. Based on experimental investigations and response surface methodology (RSM) predictions and optimization, the following key conclusions are drawn:

- LPWO was successfully extracted from lemon peel waste using steam distillation and characterized through GC-MS, FTIR, and TG/dTG analyses. GC-MS report revealed the existence of oxygenated compounds such as esters. FTIR confirmed functional moieties like O-H and C-O, indicating a significant oxygen content. The presence of hydrocarbon (C-H) groups supports LPWO’s viability as a biofuel. TG/dTG results confirmed LPWO’s high thermal stability and decomposition behaviour.
- The LPWO5 blend (5% LPWO by volume) demonstrated the highest BTE of 34.98% at a compression ratio of 18:1, exceeding diesel’s peak BTE by 1.73%. The lowest BSFC of 0.243 kg/kWh was observed for LPWO5 at maximum load and CR 18:1. Across all load conditions, LPWO blends exhibited higher EGT than diesel, along with enhancements in in-cylinder pressure and HRR.
- Among all blends, LPWO5 showed the most favourable emission profile, achieving significant reductions in CO by 59.42%, NOx by 30.99%, and smoke by 7.89% at 5.2 kW and CR 18:1 compared to diesel. However, it also resulted in increased HC and CO₂ emissions by 9.14% and 1.34%, respectively.
- RSM with CCD was used to model and optimize VCR engine responses. The optimal operating parameters predicted were a 6.51% LPWO blend ratio, 1.42 kW BP, and a CR of 18:1. Under these conditions, predicted performance outcomes were BTE of 9.59%, BSFC of 0.207 kg/kWh, and EGT of 86.75 °C. Emission values included HC at 29.32 ppm, CO at 0.028%, NOx at 415 ppm, smoke at 3.81%, and CO₂ at 2.83%.
- The optimal RSM-CCD predictions were validated experimentally using the same VCR engine test setup. The deviations between predicted and experimental values were minimal, with performance errors of 4.29% (BTE), 0.97% (BSFC), and 2.76% (EGT), and emission errors of 2.63% (HC), 3.45% (CO), 2.58% (NOx), 3.25% (smoke), and 4.71% (CO₂).
- The overall prediction error of the RSM-CCD model with desirability function analysis (DFA) was below 5%, confirming the model’s reliability for predicting and optimizing VCR engine behaviour. Consequently, the LPWO5 blend is a promising alternative to diesel for VCR engine applications, especially at a compression ratio of 18:1.

### Future recommendations

- Investigate higher LPWO blend ratios with advanced combustion strategies (EGR, optimized injection timing, multiple injection pressures).
- Explore the use of additives (oxygenates, cetane improvers, or nanoparticles) to enhance combustion efficiency and further reduce NO_x_ and HC emissions.
- Perform detailed life-cycle assessment and techno-economic analysis to validate the large-scale sustainability of LPWO biofuel.
- Extend experimental studies to multi-cylinder and modern common-rail engines for broader applicability.

## Data Availability

The datasets generated during and/or analysed during the current study are available from the corresponding author on reasonable request.

## Abbreviations

ASTM: American society for testing and materials
BP: Brake power
BTE: Brake thermal efficiency
BSFC: Brake-specific fuel consumption
bTDC: before Top dead center
BBD: Box-behnken design
CA: Crank angle
CI: Compression ignition
CO: Carbon monoxide
CR: Compression ratio
CO_2_: Carbon dioxide
CCD: Central composite design
DI: Direct injection
DFA: Desirability function approach
EGT: Exhaust gas temperature
FTIR: Fourier transform infrared spectrometry
FFD: Full factorial design
GC-MS: Gas chromatography-Mass spectrometry
HRR: Heat release rate
HC: Hydrocarbon
LPWO: Lemon peel waste oil
LPWO5: 5% Lemon peel waste oil + 95% Diesel
LPWO10: 10% Lemon peel waste oil + 90% Diesel
LPWO15: 15% Lemon peel waste oil + 85% Diesel
LPWO20: 20% Lemon peel waste oil + 80% Diesel
LPWO100: 100% Lemon peel waste oil
NOx: Nitrogen oxides
RSM: Response surface methodology
TG: Thermogravimetric analysis
dTG: Derivative thermogravimetric analysis
VCR: Variable compression ratio
